# Systematic review and meta-analysis of the effects of air pollution exposure on nasal mucosal immune-inflammatory markers in experimental animal models of AR

**DOI:** 10.3389/fphar.2026.1870023

**Published:** 2026-07-16

**Authors:** Zhuo Pan, Zeyi Lv, Xinrong Li

**Affiliations:** Hospital of Chengdu University of Traditional Chinese Medicine, ChengDu, China

**Keywords:** air pollutants, allergic rhinitis, animal models, immune response, inflammatory markers, meta-analysis, systematic review

## Abstract

Although air pollution is associated with the onset and exacerbation of allergic rhinitis (AR), epidemiological studies alone cannot reveal the tissue-level immune mechanisms because factors such as the strength of exposure, duration of exposure, presence of co-pollutants and individual susceptibility are all difficult to control. Experiments on animals have provided support for research on the immune response in the nasal mucosa under controlled conditions. A systematic review and meta-analysis were conducted to quantify changes in nasal mucosal immune-inflammatory markers and to assess the strength of mechanistic evidence from various experiments. According to PRISMA, PubMed, Web of Science, Embase and the Cochrane Library were searched from the beginning until December 2025. Eighteen eligible rodent studies were included. As the primary outcomes, type 2 inflammation-related markers were selected because they had been reported frequently and were biologically central to asthma (asthma/AR), such as eosinophils, IL-4, IL-5, IL-13 and OVA-specific IgE. Other markers included ZO-1, NLRP3, IL-1β, IL-17, IL-33, IFN-γ, neutrophils, lymphocytes and total IgE, and were used as secondary or exploratory outcomes. Pooled effects were expressed as standardised mean differences (SMDs) with 95% confidence intervals, and a random-effects model was used. Exposure to air pollutants was associated with an increase in eosinophils, elevated IL-4, IL-5, IL-13, and OVA-specific IgE; thus, a type 2 inflammatory response had been enhanced. A few studies have also indicated that there may be defects in the epithelial barrier and activation of the innate immune system, as shown by reduced ZO-1 and increased IL-1β/NLRP3. The available evidence did not show a consistent effect on IFN-γ expression. Due to considerable heterogeneity and a lack of mechanistic markers for some other reasons, these results should be interpreted cautiously and are not considered definitive causal pathways.

**Systematic Review Registration:** Identifier: CRD420251265574.

## 1 Introduction

Allergic Rhinitis (AR) is an IgE-mediated inflammation of the nasal mucosa. The classic symptoms are a stuffy nose, itching, sneezing, a runny nose, swelling and excessive mucus (Galli et al., 2008; Wallace et al., 2008). Type 2 immune inflammation in allergic rhinitis (AR) includes eosinophil infiltration, allergen-specific IgE production, and an increase in IL-4, IL-5, and IL-13. However, AR is not limited to a simple Th1/Th2 imbalance; epithelial barrier dysfunction, innate immune activation, Th17-related responses, epithelial alarmins and innate lymphoid cell pathways may also be involved in the initiation and progression of the disease (Nur Husna et al., 2021; Stanbery et al., 2022).

Fine particulate matter (PM2.5), ozone, sulphur dioxide, nitrogen dioxide, diesel exhaust particles and other air pollutants in the ambient environment can trigger or exacerbate AR and other allergic diseases (Luo P. et al., 2023; Agache et al., 2024; Pan et al., 2023; Lu et al., 2021). However, the results of human epidemiological studies are not entirely in agreement. Some cohort studies have found a relationship; others have not determined whether PM exposure is associated with AR risk (To et al., 2020; Pindus et al., 2016). The reasons for this discrepancy may include differences in exposure assessment, co-pollutant profiles, population susceptibility, outcome definitions and residual confounding.

Therefore, animal studies are still required in the absence of extensive human data. Human studies can only identify population-level associations and do not consider factors such as pollutant dose, exposure time and path, allergen sensitization history, etc. Repeatedly sample nasal mucosal tissue for clinical research is also not permitted. On the other hand, animal models can be used to conduct standardised allergen sensitisation and controlled pollutant exposure experiments to study changes in nasal mucosal immune cells, cytokines, epithelial barrier proteins, immunoglobulins and inflammasome-related markers under similar experimental conditions.

Therefore, in this paper, we will focus on immune-inflammatory indicators that reflect how pollutants damage the body and lead to allergic rhinitis (AR). The main results were selected as those that had been reported most frequently and were biologically representative of a type 2 allergic response, including eosinophils, IL-4, IL-5, IL-13, and OVA-specific IgE. Markers of epithelial barrier integrity, innate immune activation, Th17/alarmin pathways and Th1 modulation were also extracted, but they were considered secondary or exploratory outcomes when supported by only a small number of studies. The above hierarchy of results was used to avoid over-interpretation of sparse mechanistic data.

Another is that it is uneven. Existing animal studies differ in the type of pollutant, duration of exposure, concentration, route of exposure, animal strain, sensitisation protocol, tissue sampling site and detection method. And Exposure Time Matters. Animals have shown various immune and inflammatory reactions at different times after being exposed to pollutants. Li and others have linked the above differences with oxidative stress and changes in allergy symptoms (Luo X. et al., 2023). Most studies have also used single-pollutant exposure models; however, air pollution in practice is a complex mixture, and PM2.5 can contain adsorbed metals, organic compounds, and gaseous co-pollutants (Glencross et al., 2020; Li et al., 2024b). Therefore, the collection of data is not only to obtain a general idea but also to find any deficiencies or deficiencies in the supporting evidence.

Assuming that exposure to air pollutants is likely to worsen allergic rhinitis (AR) in animal models by increasing type 2 immune inflammation, we further put forward that the degree of this effect will vary based on different pollutants and lengths of exposure. Based on the above, the three main purposes of this systematic review and meta-analysis were set as follows: first, to precisely quantify the effects of air pollutant exposure on essential immune-inflammatory indicators that have received significant attention in previous studies; second, to determine whether these effects vary based on factors such as the type of pollutant, route of exposure, duration of exposure and specific animal strains used in research; finally, to differentiate among the mechanistic interpretations directly supported by the included studies and those that should be considered as hypotheses for future research, thereby providing clear directions for future study.

## 2 Methods

### 2.1 Search strategy

To conduct an all-encompassing exploration of related studies, a systematical search was performed in the PubMed, Web of Science, Embase and Cochrane Library databases, and the time period for each database was from its start date until December 2025. Both subject terms (e.g., MeSH/Emtree) and free-text words were employed to a certain extent in the search strategy. First, a large-scale search was performed using electronic retrieval methods, and then, through a careful step of manually screening the reference lists of the eligible articles, all relevant studies were comprehensively and exhaustively included. The complete search formula can be found in Supplementary Table S1. In case we overlooked anything, we have also read some related comments and references for the research papers we have selected.

According to the 2020 PRISMA statement on systematic reviews and meta-analyses, this study has been registered in PROSPERO, the international registry of systematic reviews, and was assigned the number CRD420251265574. The completed PRISMA 2020 checklist is provided in the Supplementary File S1.

### 2.2 Inclusion and exclusion criteria

Based on the PICOS criteria, the inclusion criteria were established. To make a cut, studies needed to use validated animal models of AR and compare animals exposed to pollutants with a control group that was either not exposed to pollutants or exposed to clean air and a vehicle. Accepted exposures included the above-mentioned major ambient air pollutants: PM2.5, PM10, O3, DEP, SO2 and NO2. Researchers provided the above exposures by inhalation, intranasal instillation and other explicit means. We looked for studies that measured quantitative immune-inflammatory markers in the nasal mucosa, nasal lavage fluid, serum and other relevant samples. The primary results we tracked were eosinophils, IL-4, IL-5, IL-13 and OVA-specific IgE. Secondary or exploratory endpoints included total IgE, neutrophils, macrophages, lymphocytes, IFN-γ, IL-17, IL-25, IL-33, TNF-α, IL-1β, ZO-1 and NLRP3.

In a systematic way, some research has been excluded for various reasons. First, any research that did not include a control group at the same time was excluded because it would be difficult to determine the cause. Similarly, studies that did not establish or validate an animal respiratory (AR) model were also excluded because they were not suitable for *in vivo* physiological experiments. Research that uses non-mammalian species, *in vitro* systems or *ex vivo* preparations has also been excluded because these designs cannot fully replicate the complexity of whole-organism exposure. In addition, studies were excluded if they did not measure a particular environmental air pollutant or used exposure conditions that could not isolate the effect of that pollutant from other factors. In addition to experimental design, some exclusions were made to the review, commentary and non-original research sections because they did not provide primary empirical data. Studies without convertible quantitative data, that is, those without measurable outcomes or statistical analyses, were also excluded, along with any for which full-text access could not be obtained, ensuring transparency and reproducibility of the final dataset.

### 2.3 Data extraction

Remove duplicates, and then the two reviewers independently screen the titles, abstracts and full texts to extract data in a fixed form. Extracted information includes the first author, publication year, animal species and strain, sex, sample size, AR model method, pollutant type, exposure route, concentration, frequency, duration, sampling site, detection method and outcome data. A talk or a meeting with a third party was held to resolve it. Graph-only data were reported, and the values were extracted using WebPlotDigitizer and independently verified by two reviewers.

When studies did not directly report standard deviations, they were converted from standard errors, confidence intervals, interquartile ranges or ranges according to the above method (Hozo et al., 2005). All Conversions have been recorded. As some studies have reported multiple concentrations, time points or anatomical sampling sites, a fixed rule was set to select one estimate per study for the primary analysis. This rule selected the strongest reported effect under comparable experimental conditions. However, knowing that sucnowledged it as a potential source of selection bias and subsequently applied a prioritization approach, it may have inadvertently inflated the aggregated effect size; therefore, we have also conducted rigorous sensitivity analysis to assess the effect of this on the overall results.

### 2.4 Characteristics of included studies

The event that there is a bias due to the selection of the most robustly reported effect, an additional sensitivity analysis was performed on the primary outcomes using another selection criterion. If several time points or different exposure levels were available, we selected the earlier one or the one with a lower effective concentration for a more conservative and extended analysis. Then, we compared the direction and size of all the primary endpoints with those in the main analysis to verify their consistency and stability.

### 2.5 Animal models and demographics

A meta-analysis of 18 preclinical studies on 297 AR model animals included 149 animals in the control group and 148 in the exposure group. The included studies used mice, rats and guinea pigs, and most of the models were mice and rats. BALB/c mice, C57BL/6 mice, Sprague-Dawley rats, Norway rats and Hartley guinea pigs were used. Most of the previous research has used ovalbumin sensitization; only a few studies have employed ragweed pollen or house dust mite sensitization. As shown in the included studies, there were many differences in model species, sensitisation protocols, pollutants, exposure routes, exposure durations, sampling sites and outcome measurements; therefore, a significant methodological and biological heterogeneity is expected. Therefore, the pooled estimates and the results of the subgroups were also considered in the interpretation.

## 3 Results

### 3.1 Study selection

**FIGURE 1:**
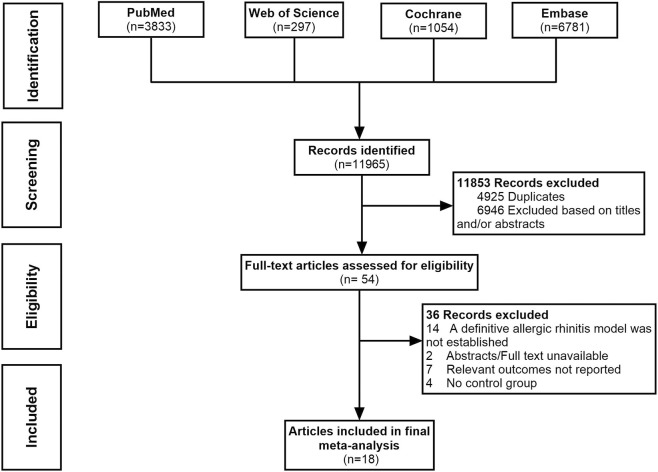
Flow chart of study selection.

The whole research screening process is shown in Figure 1, and when searching for related literature, I have used four famous databases to systematically collect as many as 11,965 relevant records so far. Then, we performed duplicate removal on the retrieved literature and excluded 4,925 duplicate documents. Next, we looked at the titles and abstracts again and removed another 6,946 that did not meet our requirements. The remaining 94 documents were added to the full-text evaluation stage, and after reading through these 94 documents, we found that 54 met the initial inclusion requirements. However, we did not stop there; we continued to investigate and excluded another 36 documents. Here’s why: 14 documents did not establish an allergic rhinitis model, so they did not meet our requirement for accuracy in the model; 2 documents could not provide the full text or abstract, and the missing information made it difficult to include them in the analysis; 7 documents did not report the outcome indicators relevant to the research and lacked key data; 4 documents did not establish a control group and thus did not meet the control condition of scientific research. After all that work, only 18 good papers were left. These provided the foundation for our Meta-analysis.

### 3.2 Characteristics of included studies

Among the 18 studies, a high proportion of them used ovalbumin (OVA) solution for sensitization, with 15 of these cases. In addition, the three studies selected ragweed pollen and dust mites for sensitisation. In total, all of the above studies have reported 17 different immunoinflammatory markers. The above markers can be divided into several categories according to the immune system. First, there are immunoglobulin profiles, and in four studies, IgE is examined; in 12, OVA-IgE is used. Next are cytokine levels, and a total of 36 studies have been conducted to examine them: 8 studies on IL-4, 6 on IL-5, 7 on IL-13, 3 on IL-17, 2 on TNF-α, 7 on IFN-γ, 3 on IL-1β, and 3 combining IL-25 and IL-33. Next, there are counts of inflammatory cells; eosinophils (Eos) have been found in 12 studies, neutrophils (Neu) have been reported in four, and macrophages (Mac) and lymphocytes (Lym) have both been mentioned a total of three times. Epithelial barrier proteins were also studied, and ZO-1 had been reported in three studies. Finally, there are inflammasome components, and NLRP3 has been studied in the three papers. Because the research sets for these different markers are not the same, to ensure the accuracy and reliability of our analysis results, we will need to perform independent Meta-analyses for each marker (or closely related groups of markers). When it comes to the overall evaluation of the full-text, 18 studies (Fukuoka et al., 2016; Guo et al., 2017; Iijima et al., 2001; Iijima and Kobayashi, 2004; Jung et al., 2021; Li et al., 2021; Li et al., 2024a; Li et al., 2025; Li et al., 2019; Park et al., 2026; Piao et al., 2021; Sun et al., 2021; Sun et al., 2023; Wagner et al., 2002; Wang et al., 2017; Ye et al., 2022; Zhang et al., 2025; Zhang et al., 2023)met all the inclusion criteria and were successfully included in the Meta-analysis. The particular number of studies (k) and the total number of animals (N) per marker are listed in the following table and subsequent sections. A summary of the main features of the included studies can be found in the Supplementary Table S3.

### 3.3 Risk bias evaluation

The two independent reviewers used the inclusion and exclusion criteria to work separately and agreed on the list of included articles. They did not have a fight. The level of strength for the chosen studies in this way was evaluated using the SYRCLE tool (Hooijmans et al., 2014). Ten attributes of each included study were randomly assessed to determine the level of bias: random sequence generation, similarity of baseline characteristics, allocation concealment, randomisation of treatment, blinding of implementers, randomisation of outcome assessment, blinding of outcome assessors, completeness of outcome data, and selective reporting of outcomes. Based on the evaluation results, all the studies were classified as having a “low risk”, “high risk” or “uncertain risk”. If there were any differences, a small group could be formed to discuss them or contacted directly with the other party.

**FIGURE 2:**
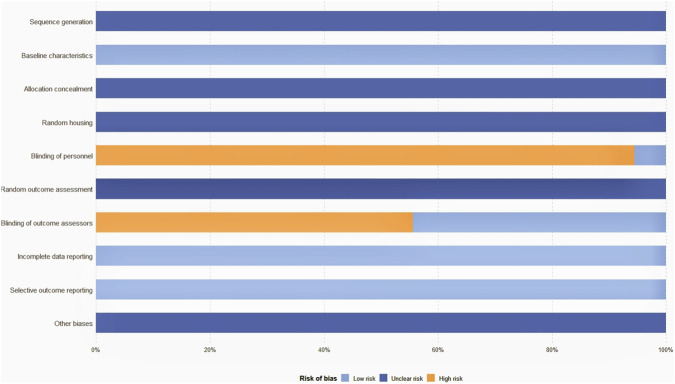
Summary of risk of bias assessment for included studies.

Overall, the risk of bias in the studies is only moderate. Most of the studies have performed selective reporting and baseline comparability well, so they are considered low risk; however, only a few studies have described the random sequence generation process, and very few have reported allocation concealment. Blinding of the experimental application and outcome assessment varied among the reported cases. Given the above circumstances, most of the studies have been rated as having an “unclear risk” or a “high risk” in the above aspects, suggesting that these studies may be prone to selection bias, implementation bias and measurement bias. Detailed evaluation results are shown in Figure 2 and Supplementary Table S4.

### 3.4 Statistical analysis

Continuous outcomes were presented as standardised mean differences (SMDs) with 95% confidence intervals (CIs). Since it was expected that the true effects varied among different animal species, pollutant types, exposure routes, concentrations, durations and detection methods, a random-effects model was employed as the main model. Coefficients Q and I^2^ were used to test the presence of heterogeneity. When there was significant heterogeneity, the pooled SMDs were interpreted as the average effect under various conditions rather than as precise generalisations.

Perform subgroup analysis based on the categories of pollutants, routes and durations of exposure, and species of animals. Cross-subgroup analyses of pollutant type and exposure duration were performed for some main outcomes where sufficient data were available. Main-text subgroup interpretation was limited to subgroups supported by at least two independent studies, and single-study subgroup results were moved to the Supplementary Material and did not support meta-analytic or mechanistic conclusions. Detailed numerical results for secondary and exploratory outcomes (e.g., IL-17, IL-33, total IgE, neutrophils, lymphocytes, ZO-1, NLRP3, IL-1β) are provided in the Supplementary Material. The main text only reports the direction of the effect and the number of supporting studies; due to a small number of studies and high heterogeneity, pooled estimates are not practical for interpretation.

If the number of studies is large enough, Begg’s and Egger’s tests for publication bias will be used. As these tests have low power due to the small number of studies (fewer than ten), their results should be interpreted cautiously. Leave-One-Out Sensitivity Analysis was conducted to examine the effect of individual studies. An alternative selection of data was also conducted for the primary outcomes to assess the stability of the results under a different selection rule for the strongest-effect data point; that is, among several data points, the earliest eligible time point or the lowest effective concentration was chosen.

## 4 Meta-analysis conclusion

### 4.1 Primary outcome

**FIGURE 3:**
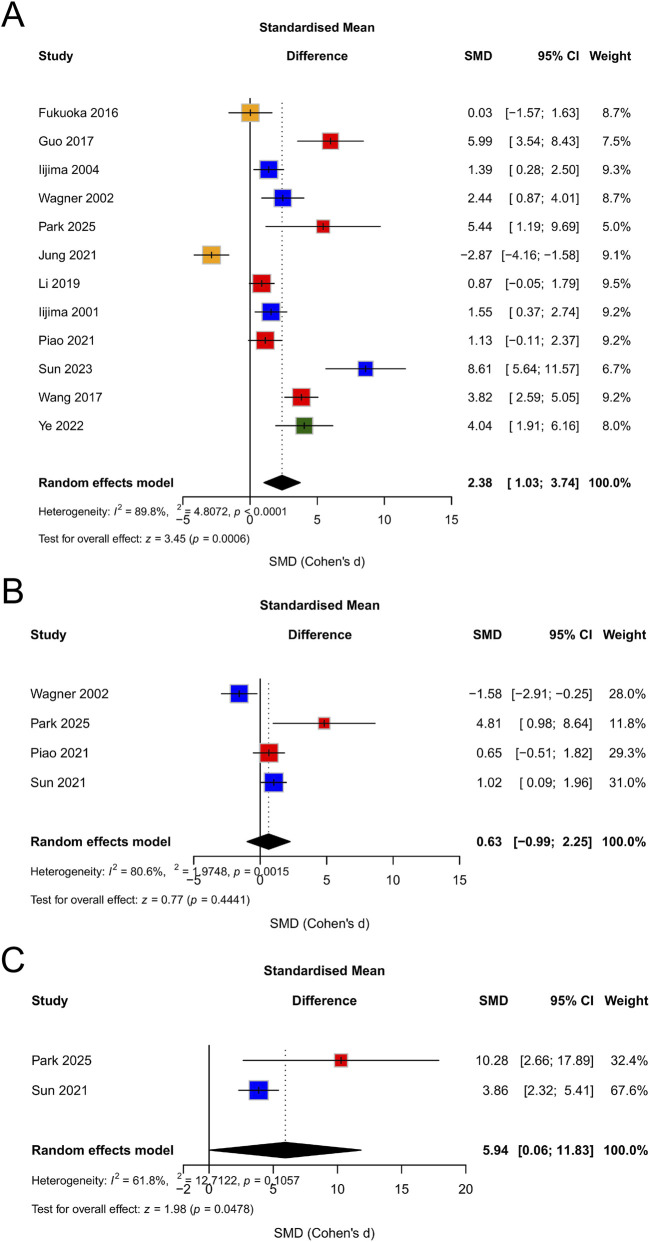
Forest plot of the effect of Air Pollution on **(A)** eosinophil counts, **(B)** neutrophil counts, **(C)** lymphocyte counts.

**FIGURE 4:**
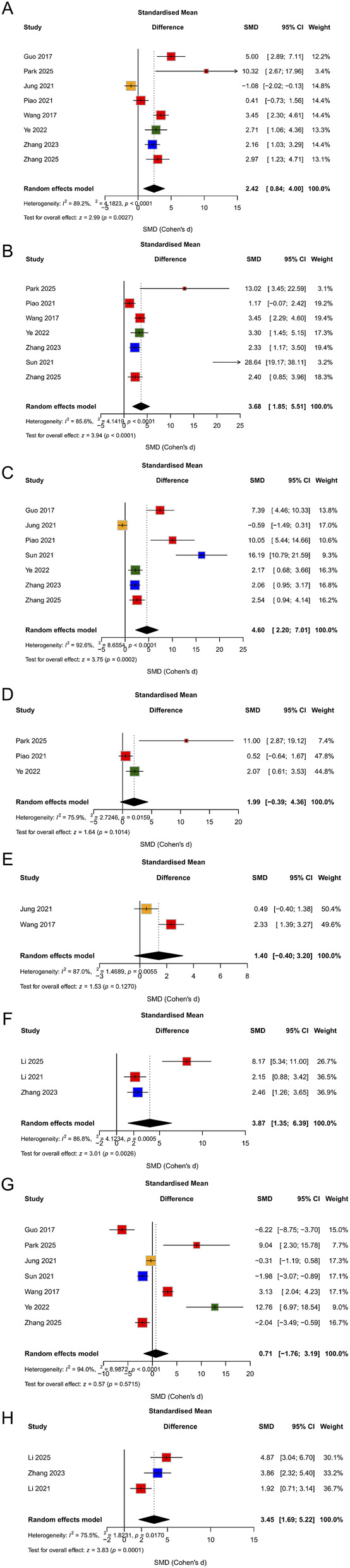
Forest plot of the effect of Air Pollution on **(A)** IL-4, **(B)** IL-5, **(C)** IL-13, **(D)** IL-17, **(E)** IL-33, **(F)** IL-1β, **(G)** IFN-γ, **(H)** NLRP3.

**FIGURE 5:**
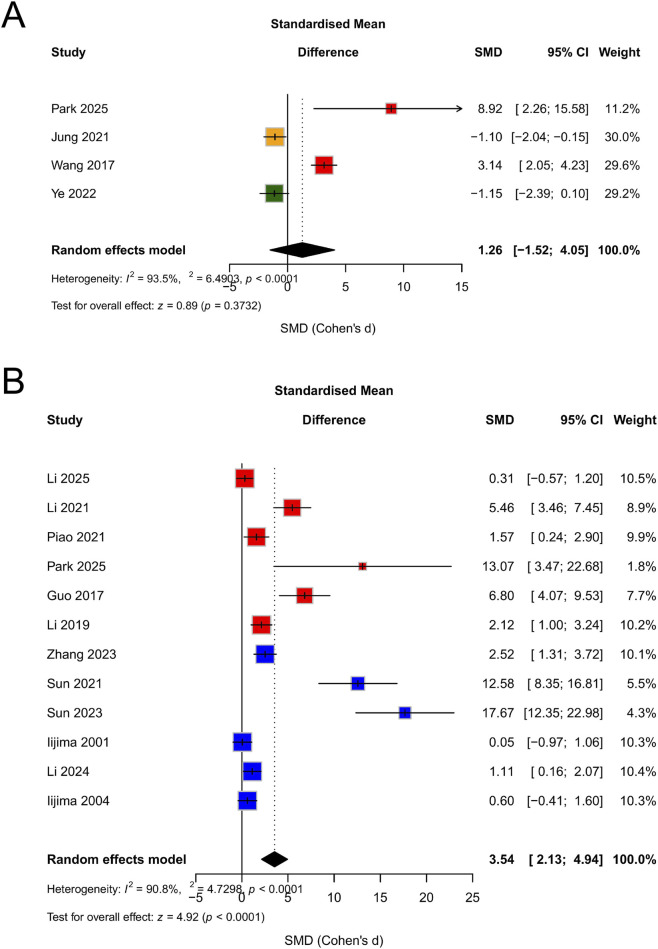
Forest plot of the effect of Air Pollution on **(A)** IgE, **(B)** ova-IgE.

Exposure to air pollutants is associated with an increase in the main type 2 inflammation-related endpoints. Eosinophil levels were elevated in all 12 comparisons [SMD = 2.38, 95% CI: 1.04–3.74, p = 0.0006, I^2^ = 89.8%] (see Figure 3A for more details), and Th2-related cytokines were also raised, such as IL-4 [n = 8, SMD = 2.42, 95% CI: 0.84–4.00, p = 0.0027, I^2^ = 89.2%] (Figure 4A), IL-5 [n = 7, SMD = 3.68, 95% CI: 1.88–5.51, p < 0.0001, I^2^ = 85.6%](Figure 4B),and IL-13 [n = 7, SMD = 4.60, 95% CI: 2.20–7.01, p = 0.0002, I^2^ = 92.6%] (Figure 4C). OVA-specific IgE was also increased [n = 12, SMD = 3.54, 95% CI: 2.13–4.94, p < 0.0001, I^2^ = 90.8%] (Figure 5B). In short, air pollutants increase the extent of type 2 allergic inflammation. However, the high I^2^ values also tell us that we have averaged the effects of many different animal models; there is no single story here.

### 4.2 Exploratory results

**FIGURE 6:**
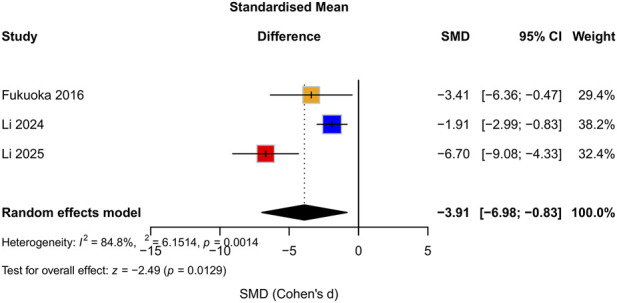
Forest plot of the effect of Air Pollution on ZO-1

Several secondary or exploratory markers provided the first mechanistic information. IL-1β was elevated in three studies [SMD = 3.87, 95% CI: 1.35–6.39, p = 0.0026, I^2^ = 86.8%] (Figure 4F),NLRP3 was elevated in three studies [SMD = 3.45, 95% CI: 1.69–5.22, p = 0.0001, I^2^ = 75.5%] (Figure 4H), and ZO-1 was reduced in three studies [SMD = −3.91, 95% CI: −6.98 to −0.83, p = 0.0129, I^2^ = 84.8%] (Figure 6). Lymphocyte infiltration was reported in only two studies [SMD = 5.94, 95% CI: 0.06–11.83, p = 0.0478, I^2^ = 61.8%] (see Figure 3C). However, these findings originate from small sample sizes and the results are quite inconsistent; thus, at present, they only offer us a hint rather than a conclusive answer. The pooled effect for IFN-γ[n = 7, SMD = 0.71, 95% CI: −1.76 to 3.19, p = 0.5715, I^2^ = 94%], neutrophils [n = 4, SMD = 0.63, 95% CI: −0.99 to 2.25, p = 0.4441, I^2^ = 80.6%], IL-17 [n = 3, SMD = 1.99, 95% CI: −0.39 to 4.36, p = 0.1014, I^2^ = 75.9%], IL-33 [n = 2, SMD = 1.40, 95% CI: −0.40 to 3.20, p = 0.1270, I^2^ = 87%], and total IgE [n = 4, SMD = 1.26, 95% CI: −1.52 to 4.05, p = 0.3732, I^2^ = 93.5%] were not statistically significant. Therefore, based on the available evidence, it cannot be concluded that air pollution affects IFN-γ expression consistently, nor has a change in Th1 regulation been identified.

### 4.3 Subgroup analysis

As most of the results showed relatively large differences, subgroup analysis was performed to determine why there were differences, rather than confirming the specific effects of individual pollutants. Given that there were few studies in a particular subgroup, the above analyses should be taken into account cautiously.

By pollutant type, O3 exposure was associated with an increase in eosinophils [n = 4, SMD = 3.03, 95% CI: 1.03–5.03, p = 0.0001, I^2^ = 85.8%] and OVA-specific IgE [n = 6, SMD = 4.00, 95% CI: 1.77–6.24, p < 0.0001, I^2^ = 93.4%]. PM2.5 was the most frequently studied pollutant, and it was associated with all five types of type 2 inflammatory markers, including IL-13 [n = 3, SMD = 6.30, 95% CI: 1.78–10.83, p = 0.0006, I^2^ = 86.6%], eosinophils [n = 5, SMD = 3.04, 95% CI: 1.18–4.89, p < 0.0001, I^2^ = 86.1%], IL-4 [n = 5, SMD = 3.28, 95% CI: 1.28–5.27, p < 0.0001, I^2^ = 84%], IL-5 [n = 4, SMD = 2.66, 95% CI: 0.96–4.37, p = 0.0089, I^2^ = 74.1%], and OVA-specific IgE [n = 6, SMD = 3.35, 95% CI: 1.38–5.32, p < 0.0001, I^2^ = 88.2%]. That said, these numbers do not indicate that PM2.5 has a stronger effect than other pollutants; the groups varied in how many studies examined each one, how exposures were measured, and at what levels of heterogeneity. Pollutants that have only been studied in a single paper (including some related to DEP) have their subgroup results in the Supplementary Material and are not used to derive mechanistic conclusions here.

By exposure route, whole-body inhalation was associated with increased eosinophils [n = 7, SMD = 3.14, 95% CI: 1.66–4.63, p < 0.0001, I^2^ = 85.2%], OVA-specific IgE [n = 8, SMD = 3.97, 95% CI: 2.13–5.80, p < 0.0001, I^2^ = 92.4%], IL-4 [n = 5, SMD = 2.99, 95% CI: 1.91–4.07, p = 0.0001, I^2^ = 44.7%], IL-5 [n = 4, SMD = 4.80, 95% CI: 1.67–7.93, p < 0.0001, I^2^ = 89.9%], and IL-13 [n = 5, SMD = 4.84, 95% CI: 2.31–7.37, p < 0.0001, I^2^ = 88.7%]. Intranasal administration was associated with increased IL-5 [n = 3, SMD = 3.03, 95% CI: 0.39–5.68, p = 0.0030, I^2^ = 82.8%] and OVA-specific IgE [n = 4, SMD = 3.01, 95% CI: 0.32–5.71, p < 0.0001, I^2^ = 89%]. Results from a single study were moved to the Supplementary Table S6.

Short-term exposure (≤7 days) was associated with increased eosinophils due to an extended exposure period [n = 5, SMD = 2.04, 95% CI: 0.53–3.55, p = 0.0044, I^2^ = 73.6%]. Medium-term exposure (8–30 days) was associated with increased IL-13 [n = 5, SMD = 3.61, 95% CI: 1.03–6.20, p < 0.0001, I^2^ = 92.2%], IL-4 [n = 6, SMD = 2.05, 95% CI: 0.30–3.81, p < 0.0001, I^2^ = 91.3%], IL-5 [n = 4, SMD = 2.35, 95% CI: 1.39–3.32, p = 0.0750, I^2^ = 56.6%], and OVA-specific IgE [n = 5, SMD = 2.38, 95% CI: 1.20–3.57, p = 0.0026, I^2^ = 75.6%]. Long-term exposure (>30 days) was associated with increased eosinophils [n = 3, SMD = 3.41, 95% CI: 0.59–6.22, p < 0.0001, I^2^ = 90.4%] and OVA-specific IgE [n = 4, SMD = 6.55, 95% CI: 2.34–10.76, p < 0.0001, I^2^ = 95.7%]. The above results indicate a possible time-dependent pattern, but they should be treated as hypothesis-generating; high heterogeneity and a small number of studies were observed in some time windows.

According to the data at the level of animal species, some changes were observed in several types of Type 2 inflammatory markers in mice and rats; however, the guinea pig subgroup did not show the same effect. The differences in the above species are probably due to a combination of reasons, such as model-specific immune sensitivity, variations in sensitization protocols, various pollutant exposure environments, and different measurement methods for the outcome. Since some subgroup estimates for specific animal strains were obtained from a very small number of studies, the results of single studies have been limited to Supplementary Table S8 and deliberately excluded from the foundation of any general conclusions.

Cross-analysis of pollutant type and exposure duration showed that PM2.5 combined with medium-term exposure was associated with an increase in OVA-specific IgE [n = 3, SMD = 3.12, 95% CI: 0.92–5.31, p = 0.0029, I^2^ = 82.9%], IL-4 [n = 4, SMD = 2.86, 95% CI: 0.93–4.79, p = 0.0001, I^2^ = 85.7%], IL-5 [n = 3, SMD = 2.35, 95% CI: 0.95–3.76, p = 0.0318, I^2^ = 71%], eosinophils [n = 3, SMD = 3.48, 95% CI: 0.97–5.99, p = 0.0003, I^2^ = 87.6%], and IL-13 [n = 3, SMD = 6.30, 95% CI: 1.78–10.83, p = 0.0006, I^2^ = 86.6%]. O3 combined with medium-term or prolonged exposure was associated with an increase in OVA-specific IgE and eosinophils, but our subgroup sizes were small and there was a considerable difference. Therefore, we should consider the above results to be indicators that require further investigation and are not yet definite evidence of specific pollutants.

### 4.4 Subgroup forest plot summary

Additional forest plots were constructed to summarise subgroup analyses by pollutant type, exposure route, exposure duration, animal species and pollutant-by-duration combinations (Supplementary Figures S1-S5). The above are the pooled SMDs, 95% CIs and heterogeneity estimates for each subgroup. They have presented patterns and put forward hypotheses; they have not determined the reasons for the change.

The subgroup plots generally show that the effect estimates are different in the experimental conditions. PM2.5 and O3 were the most frequently occurring pollutants, and several types of type 2 inflammatory markers were associated with them; however, due to disparities in the number of studies, exposure conditions and measurement indices, direct comparisons among different pollutants have not been widely conducted. Therefore, any differences in PM2.5 and O3 should be considered as possible biological phenomena that still need to be verified by standardised animal experiments directly comparing them or observing mixtures of pollutants.

### 4.5 Publication bias and sensitivity analysis

Begg’s and Egger’s tests for publication bias were performed where feasible (Supplementary Table S10). However, these tests are not very reliable when there are only a few studies, and especially so if there are fewer than ten studies for a particular outcome. Therefore, we treated the results of these tests as supplementary information and did not consider them to be conclusive evidence of publication bias.

Begg’s or Egger’s tests indicated that some of the endpoints, such as OVA-specific IgE, IL-5 and IL-13, may have a small-study effect. Based on the above analysis, it can be inferred that the pooled effects for some primary outcomes may have been inflated. Given the limited research availability of other outcomes, such as NLRP3, IL-1β and ZO-1, while statistical tests show no publication bias, this does not definitively prove that no such bias exists; rather, the lack of sufficient statistical power due to small sample sizes in some studies cannot be ruled out.

**FIGURE 7:**
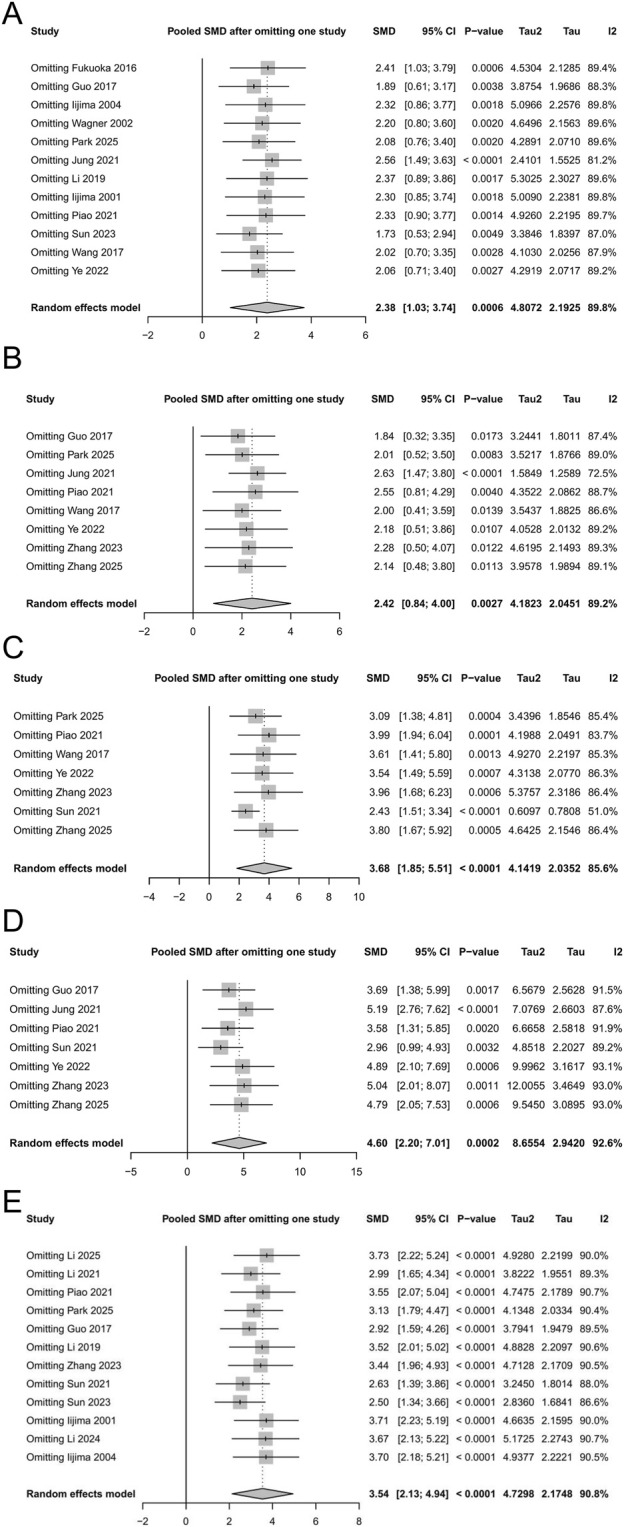
Forest plot of sensitivity analysis for the effect of Air Pollution on **(A)** eosinophil counts, **(B)** IL-4, **(C)** IL-5, **(D)** IL-13, **(E)** ova-IgE.

Leave-one-out sensitivity analyses were conducted for the main outcomes: eosinophils, IL-4, IL-5, IL-13 and OVA-specific IgE (Figure 7). When we took out each study one by one, the estimated results did not change significantly in direction; therefore, the positive results were not due to a single study. However, this analysis has not accounted for the possible selection bias caused by selecting the largest reported effect in the studies.

**FIGURE 8:**
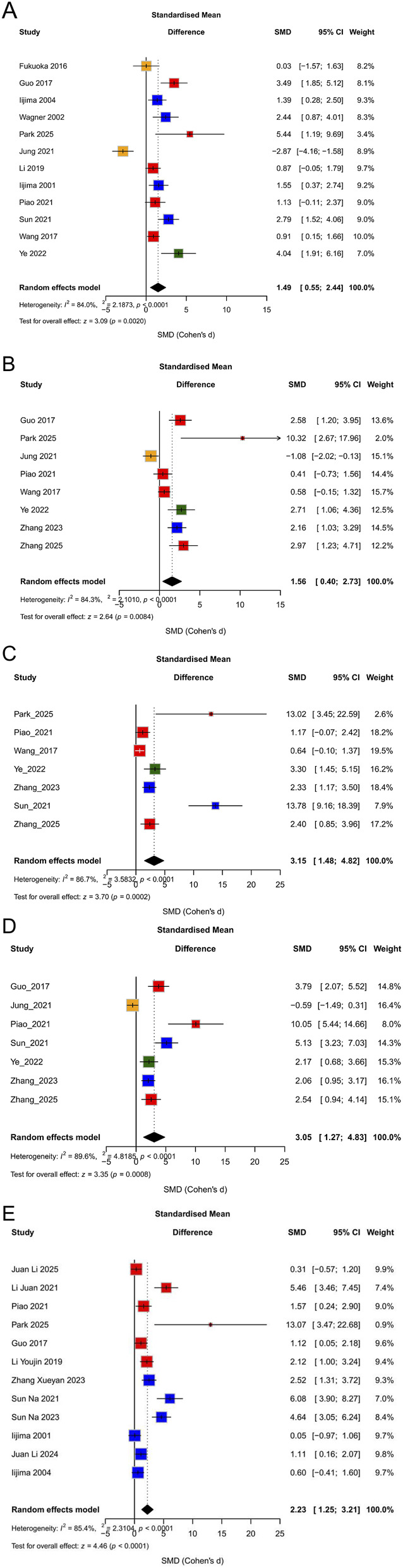
Post-hoc sensitivity analysis. **(A)** eosinophil counts, **(B)** IL-4, **(C)** IL-5, **(D)** IL-13, **(E)** ova-IgE.

Therefore, using a different rule for selecting the primary outcomes, we also performed an additional sensitivity analysis (as shown in Figure 8). In this analysis, the main estimates were compared with the results of using the earliest eligible time point, the lowest effective concentration or other pre-specified rules when multiple data points were available in the same study. Change in direction, size and statistical significance of the pooled estimates were examined to determine whether the main results were stable. If the direction or statistical significance did not differ from the main analysis, then this conclusion was also regarded with caution. The corresponding specific numerical results are shown in the Supplementary Table S11.

## 5 Conclusions of the meta-analysis

### 5.1 Principal findings

Through this comprehensive systematic review and meta-analysis, we have gathered preclinical animal data to explore the effects of air pollutant exposure on immune-inflammatory markers in allergic rhinitis (AR) models. First, people who are exposed to air pollution are more likely to have type 2 allergies. As shown in the above figure, there was an increase in eosinophils and higher levels of the cytokines IL-4, IL-5, and IL-13; at the same time, OVA-specific IgE also increased significantly; all of these indicate an enhanced allergic reaction. Evidence of epithelial barrier disruption and inflammasome activation, on the other hand, was biologically plausible but had fewer studies supporting it. Therefore, the above paths should be regarded as an organised mechanistic hypothesis based on the measured markers, rather than an established causal sequence.

The current results are in line with the existing immunology of AR. Type 2 inflammation is shown to be associated with eosinophil infiltration, IgE-mediated allergic reactions and increased Th2-related cytokines; it also occurs in nasal mucosa and presents as allergic symptoms (Galli et al., 2008; Wallace et al., 2008). However, according to the present study, air pollution-related exacerbation of AR cannot be fully attributed to a simple Th1/Th2 imbalance. Other immune and epithelial changes that may promote the progression of the disease include impaired barrier function, activation of the innate immune system, epithelial alarmins, and Th17-related responses; however, most of the evidence remains at the animal level (Nur Husna et al., 2021).

### 5.2 Type 2 allergic inflammation as the most consistent signal

Among all the results, eosinophils, IL-4, IL-5, IL-13 and OVA-specific IgE showed the most consistent direction of change. These markers are all related but not identical components of type 2 allergic inflammation. IL-4 is required for the differentiation and maintenance of type 2 immune responses and IgE class switching; IL-5 is needed to promote eosinophil maturation and recruitment; and IL-13 is associated with mucus production, epithelial changes and airway allergic inflammation (Galli et al., 2008; Wallace et al., 2008). Looking at the data, eosinophils, IL-4, IL-5, IL-13 and OVA-specific IgE all increase together in the same direction and quite reliably.

Based on the above results, air pollution can cause more allergic inflammation in the system that is already prone to AR. However, the size of the pooled effects should also be interpreted cautiously. Most of the primary outcomes showed considerable heterogeneity; thus, the absolute SMDs cannot be considered precise universal estimates. All these factors can affect the size of the observed effect: different animal species, different sensitisation protocols, various pollutants, different exposure routes, various exposure concentrations, different exposure durations, different tissue sampling sites, and different detection methods. What you can be certain of: air pollution consistently increases Type 2 inflammatory responses, but the degree of this increase varies from experiment to experiment.

### 5.3 Epithelial barrier disruption and inflammasome activation: plausible but limited evidence

Exploratory results indicated reduced ZO-1 expression and elevated NLRP3 and IL-1β in pollutant-exposed AR models. The Changes are biologically meaningful. ZO-1 is a typical tight-junction-associated protein; therefore, a decrease in its level may indicate damage to the epithelial barrier. This means the nasal lining is less protective; more allergens reach the immune system, and thus inflammation in allergic rhinitis (AR) is exacerbated (Nur Husna et al., 2021). Other scholars have also seen this trend. PM2.5 exposure leads to oxidative stress and damage of the nasal epithelium in the presence of AR (Mariani et al., 2021; Yuan et al., 2025; Li et al., 2024a).

NLRP3 and IL-1β are upstream regulators of inflammasome-mediated innate immunity, as shown by Dinarello (2018). A few animal studies have actually measured NLRP3 or IL-1β in this review to find that pollutants trigger innate inflammation in the nasal mucosa (Li et al., 2021; Li et al., 2025). However, these results only come from a few studies and are quite different from one another. Therefore, we cannot use this data to conclude that the epithelial barrier has been lost before NLRP3 is activated, or that inflammasome activation directly causes Type 2 polarisation. On the other hand, the combined results do not offer a biologically plausible mechanism for the interaction among epithelial injury, innate immune activation and type 2 inflammation during pollutant-aggravated asthma. Based on the available biomarkers, type 2 inflammation was the most consistently supported mechanistic pathway, as indicated by eosinophils, IL-4, IL-5, IL-13 and OVA-specific IgE. On the other hand, epithelial barrier disruption, inflammasome-related innate activation, Th1 modulation, and the Th17/alarmin-related pathway have been studied less frequently or show inconsistent results. Therefore, the above mechanisms were considered exploratory or hypothesis-generating rather than definite causal paths. The evidence mapping of the mechanistic interpretations is presented in the Supplementary table S12.

### 5.4 Interpretation of IFN-γ, Th1 modulation, and other immune pathways

Based on the above analysis, it can be determined that the pooled effect for IFN-γ is not statistically significant and shows relatively large heterogeneity. Therefore, the existing evidence does not provide full support for a general effect of air pollution on the expression of IFN-γ. Therefore, it cannot be concluded that there is a regulation of Th1 cells. Based on the above observations, it cannot be assumed that air pollution affects the immune system through the modification of Th1/Th2 differentiation; therefore, no such claim can be made without further investigation.

The results of IL-17, IL-25, IL-33, TNF-α, neutrophils, macrophages and lymphocytes were relatively limited due to a small number of studies or inconsistent results. These markers may still be relevant to AR pathophysiology in relation to epithelial alarmins, innate lymphoid cell responses and non-type 2 inflammatory pathways (Nur Husna et al., 2021; Stanbery et al., 2022). However, the existing animal evidence has not shown a clear direction or mechanism for these routes; thus, future research needs to use broad immune profiling and should not be limited to the traditional Th1/Th2 markers.

### 5.5 Pollutant-specific patterns and biological plausibility

Studies of particular groups have focused on PM2.5 and O3 as the pollutants to be investigated, and these were connected to alterations in several types of Type 2 inflammatory markers. PM2.5 was associated with eosinophils, Th2-related cytokines and OVA-specific IgE. It may be biologically plausible that particulate matter deposits on the nasal mucosa and carries adsorbed metals and organic compounds and other co-pollutants. The above factors may cause oxidative stress and disruption of the epithelial barrier, leading to the activation of downstream immune cells (Glencross et al., 2020; Li et al., 2023; Li et al., 2024b).

O3 may have a different immune response because it is a strong oxidant that can react with airway surface fluid and epithelial cells to cause oxidative damage to the epithelium and trigger inflammation (Wiegman et al., 2020; Bao et al., 2018). Previous experimental and epidemiological studies have linked O_3_ exposure with nasal or allergic inflammatory responses (Wang et al., 1995; Peden et al., 1995; Kim et al., 2011; Ye et al., 2024; Li et al., 2024a). Therefore, the immune patterns of PM2.5 and O3 are likely different biologically.

Based on the above results, it has not been determined whether PM2.5 is more harmful than the other pollutants. Pollutant subgroups varied in the number of studies, exposure design, concentration, route, duration and outcome measurement. Direct head-to-head comparisons of the pollutants were unavailable. In addition, most of the included animal studies used a single-pollutant exposure model, and real-world air pollution is a complex mixture. Therefore, the results for individual pollutants should be viewed as hypothesis-generating rather than as conclusive proof of pollutant superiority.

### 5.6 Time-dependent immune response patterns

Another reason for the different results of the study may be how long the people were exposed. Based on the above analysis of the effect of different durations, it can be concluded that a short exposure time mainly results in early inflammatory changes, such as eosinophil infiltration and the appearance of exploratory markers for epithelial or innate immune system activation. As we turned our attention to the medium-term observation period, a new trend appeared; that is, at this time, the levels of several types of type 2 inflammatory cytokines had changed. Both eosinophils and OVA-specific IgE increased over the long term in this study. Based on the above, it can be assumed that pollutant exposure first damages the epithelial and innate immune systems, and then progressively promotes allergen-specific type 2 immune responses over time (Li et al., 2023; Ye et al., 2022).

However, this temporal interpretation should be regarded as a working model rather than an empirically validated dynamic immune-regulatory network. The included studies varied in exposure-duration categories, pollutants, schedules of sensitisation, and sampling times. Most studies have not repeatedly measured the same immune markers at different times. Therefore, although the observed results suggest a possible time-dependent immune pattern, future animal studies with standardised long-term sampling need to be conducted to clarify the sequence of epithelial injury, innate immune activation, type 2 cytokine production, eosinophilic inflammation and IgE responses.

### 5.7 Heterogeneity, data-selection strategy, and robustness of evidence

Most of the results were quite varied. Heterogeneity is expected in preclinical environmental health studies because of various differences among the experiments, such as different animal species, allergenisation protocols, exposure concentrations, pollutant compositions, exposure routes and detection methods. Given the different experimental conditions, the pooled standardised mean differences (SMDs) should be interpreted as the average effect under these heterogeneous circumstances and are not precise estimates that can be universally applied to all animal models of AR.

Another problem with the methods is the predetermined strong-effect data selection rule in the main analysis. The highest exposure concentration, the largest absolute SMD time point or the strongest anatomical-site effect may be selected to capture biological signals, but this can also increase the pooled effect size and cause heterogeneity. Therefore, this way should not be shown as a validated standard method. A Sensitivity Analysis can be used here. Run a second analysis using a more conservative or prespecified rule to get a reality check and see whether the main findings still hold under stricter conditions. If the direction of the results changes or they no longer reach statistical significance after using the alternative, then that conclusion should be treated with more caution.

The leave-one-out sensitivity analysis showed that the general direction of our main results was not significantly affected by any single person’s study; however, we must also be aware of the deficiencies in this method. Leave-one-out analysis cannot correct for selection bias within the study caused by selecting the strongest reported effect. Therefore, both kinds of sensitivity analysis need to be carried out at the same time to determine how stable the evidence is.

### 5.8 Candidate biomarkers and translational implications

EOS, IL-13, and OVA-specific IgE may be considered candidate translational biomarkers because they were biologically relevant to AR, reported in a relatively large number of studies, and showed a consistent direction of change in the present analysis. EOS is a good indicator of cellular allergic inflammation, and IL-13 is a typical component of the central type 2 cytokine response. These markers can be used in the following animal studies and translational research to link pollutant exposure with AR exacerbation.

ZO-1, NLRP3 and IL-1β are likely to be mechanistic markers rather than established biomarkers. They are biologically plausible, but the body of evidence is small at present. In the future, one will determine whether the above indicators show the same trend across all pollutants, exposure times and AR models. Add the above indicators to symptom scores, nasal function and epithelial barrier index data, as well as environmental exposure data in clinical and population-based studies.

### 5.9 Limitations and future directions

Several deficiencies will be listed below. First, most of the outcomes were still considerably heterogeneous, and thus the precision of the pooled estimates was relatively low. Secondly, some markers and subgroups have only been supported by two or three studies. The above results offer a path for research but are not conclusive. Third, employing the strongest-effect selection rule may have inflated some of the pooled results. Given the reliability of the first set of results, a sensitivity analysis will also be conducted. Fourth, excluding non-English publications introduces language bias. Fifth, Begg’s and Egger’s tests are unreliable in the presence of a small number of studies, so a negative result here cannot be taken as evidence against publication bias (Sterne et al., 2011). Lastly, most of the previous research has used animal models and only tested a single pollutant at a time. The above arrangement cannot simulate the actual environment of a complex air pollution mixture during exposure (Glencross et al., 2020; Li et al., 2024b).

Future studies will use a more standardised AR model procedure, set up pollutant exposure protocols, select sampling time points and detection methods. Experimental Design should include head-to-head comparisons of different pollutants and mixture-based exposure models that are closer to real-world air pollution. All sorts of statistical methods, such as weighted quantile sum regression and Bayesian kernel machine regression, can be used in population-based research to find the joint and interactive effects of multiple pollutants (Carrico et al., 2015; Bobb et al., 2015). In the future, some research may be conducted on immune markers, epithelial-barrier indicators, nasal symptoms and exposure measurements to enhance the translational value of the animal studies.

Based on the synthesis of the results of this meta-analysis, it can be concluded that exposure to air pollutants is closely associated with the worsening of type 2 allergic inflammation in animal models of asthma. Evidence of epithelial barrier disruption and inflammasome activation is biologically plausible but still experimental. Due to the high heterogeneity, a small number of studies for some markers, and the dominance of single-pollutant models, the results should be regarded as preclinical evidence supporting mechanistic hypotheses rather than conclusive proof of an entire causal pathway.

## 6 Conclusion

Based on the above research, exposure to air pollutants can enhance the type 2 inflammatory response in animal models of asthma (AR), with features such as increased eosinophil infiltration, elevated Th2 cytokines, and OVA-specific IgE. Limited evidence also suggests the possible disruption of the epithelial barrier and activation of the innate immune system, as shown by ZO-1, NLRP3 and IL-1β. However, these mechanistic results are based on a small number of studies and should be regarded as exploratory.

Although the existing body of evidence has been thoroughly investigated, it has failed to demonstrate a uniform effect of air pollution on the expression of IFN-γ, and thus, any definitive conclusions regarding the regulation of Th1 cells should be drawn cautiously and, ideally, avoided until further evidence is provided. Although the differences in the effects of PM2.5 and O3 on immune responses are biologically plausible, suggesting different mechanisms of action or varying degrees of cellular damage, these results cannot be taken as definitive because the studies included in this analysis have considerable heterogeneity in design, population and methodology, and there are few opportunities for direct comparison of these pollutants. In addition, there are several methodological deficiencies in the support for these observations; first, there is considerable dispersion among the research results; second, selection bias may have occurred due to strict inclusion criteria for the data; third, a single-pollutant model is employed that does not account for the complexity of real-world exposure; and finally, there is a potential for language bias in the literature search and selection process.

To deepen our understanding of how air pollution induces AR pathology and to strengthen the translational value of preclinical research for environmental health protection, the following studies will be strategically focused on in the future. First, a standardised exposure protocol should be used to ensure the uniformity and replicability of the studies for more reliable comparison of results. Use other ways to select the data for a sensitivity analysis and check if there is any bias in the results of the research. Additionally, to address the combined impact of several pollutants in the environment more realistically, a mixture-based pollutant model can be employed. Finally, through in-depth human and translational studies of the candidate biomarkers, specific information will be obtained on how air pollution causes AR at the molecular level and the reliability of preclinical data for new prevention measures will be improved. Based on the above analysis in the near future, researchers will systematically tackle these problems to deepen our understanding of this serious public health issue and promote targeted prevention and control.

## Data Availability

The original contributions presented in the study are included in the article/Supplementary Material, further inquiries can be directed to the corresponding author.
