# Supplementary material for: Systematic review and meta-analysis of the effects of air pollution exposure on nasal mucosal immune-inflammatory markers in experimental animal models of AR
Source: Front Pharmacol. 2026 Jul 16;17:1870023. doi: 10.3389/fphar.2026.1870023 (PMC13422168; doi:10.3389/fphar.2026.1870023)
Supplement: Supplementary file 1 [file Supplementaryfile1.zip › Supplementary file 1/Supplementary Table 1.docx]

**Embase**

'aerial pollution' OR 'aerogenic pollution' OR 'air contamination' OR 'air pollutioning' OR 'air-borne pollution' OR 'airborne pollution' OR 'atmosphere pollution' OR 'atmospheric pollution' OR 'polluted air' OR 'polluted atmosphere' OR 'pollution, air' OR 'air pollution'OR particulate matter OR PM2.5 OR PM10 OR nitrogen dioxide OR NO2 OR NO OR ozone OR O3 OR sulfur dioxide OR SO2 OR carbon monoxide OR CO

'allergic rhinopathy' OR 'atopic rhinitis' OR 'eosinophil rhinitis' OR 'eosinophile rhinitis' OR 'eosinophilic rhinitis' OR 'eosinophilous rhinitis' OR 'rhinitis allergica' OR 'rhinitis atopica' OR 'rhinitis eosinophila' OR 'rhinitis, allergic' OR 'allergic rhinitis'

"inflammatory marker" OR "inflammatory mediator" OR cytokine OR interleukin OR "IL" OR tumor necrosis factor OR TNF OR TNF-alpha OR interferon OR IFN OR histamine OR leukotriene OR eosinophil OR eosinophilic OR eosinophils OR mast cells OR lymphocytes OR neutrophils OR macrophages OR nasal mucosa OR nasal secretions OR IgE OR CRP OR Fibrinogen OR NLRP3

'animal population groups' OR 'Animalia' OR 'animals' OR 'Metazoa' OR 'metazoan' OR 'metazoans' OR 'metazoon' OR 'animal'

'mice' OR 'Mus (genus)' OR 'newborn mice' OR 'mouse'

History  Save |Delete |Print view |Export |EmailCombineusingAndOr

Collapsev

#16

#5 AND #8 AND #9 AND #15

6,779

#15

#12 OR #13 OR #14

37,434,277

#14

mouse:ab,ti OR mice:ab,ti OR 'newborn mice':ab,ti OR (mus:ab,ti AND genus:ab,ti)

2,144,806

#13

'mouse'/exp OR 'mouse'

2,688,627

#12

#10 OR #11

37,383,068

#11

'animal population groups':ab,ti OR animalia:ab,ti OR animals:ab,ti OR metazoa:ab,ti OR metazoan:ab,ti OR metazoans:ab,ti OR metazoon:ab,ti OR animal:ab,ti

1,705,700

#10

'animal'/exp OR 'animal'

37,338,992

#9

'inflammatory marker':ab,ti OR 'inflammatory mediator':ab,ti OR 'cytokine':ab,ti OR il:ab,ti OR tnf:ab,ti OR 'tumor necrosis factor':ab,ti OR 'interferon':ab,ti OR ifn:ab,ti OR 'histamine':ab,ti OR 'leukotriene':ab,ti OR eosinophilic:ab,ti OR 'eosinophil':ab,ti OR 'mast cell':ab,ti OR 'lymphocyte':ab,ti OR 'neutrophil':ab,ti OR 'macrophage':ab,ti OR 'nose mucosa':ab,ti OR 'nasal secretions':ab,ti OR 'immunoglobulin e':ab,ti OR 'c reactive protein':ab,ti OR 'fibrinogen':ab,ti OR 'nlrp3':ab,ti OR ige:ab,ti

2,163,196

#8

#6 OR #7

70,657

#7

'allergic rhinitis':ab,ti OR 'allergic rhinopathy':ab,ti OR 'atopic rhinitis':ab,ti OR 'eosinophil rhinitis':ab,ti OR 'eosinophile rhinitis':ab,ti OR 'eosinophilic rhinitis':ab,ti OR 'eosinophilous rhinitis':ab,ti OR 'rhinitis allergica':ab,ti OR 'rhinitis atopica':ab,ti OR 'rhinitis eosinophila':ab,ti OR 'rhinitis, allergic':ab,ti

37,491

#6

'allergic rhinitis'/exp

66,091

#5

#3 OR #4

8,915,479

#4

'particulate matter':ab,ti OR pm2.5:ab,ti OR pm10:ab,ti OR 'nitrogen dioxide':ab,ti OR no2:ab,ti OR no:ab,ti OR o3:ab,ti OR ozone:ab,ti OR 'sulfur dioxide':ab,ti OR so2:ab,ti OR 'carbon monoxide':ab,ti OR co:ab,ti

8,757,519

#3

#1 OR #2

260,783

#2

'air pollution':ab,ti OR 'aerial pollution':ab,ti OR 'aerogenic pollution':ab,ti OR 'air contamination':ab,ti OR 'air pollutioning':ab,ti OR 'air-borne pollution':ab,ti OR 'airborne pollution':ab,ti OR 'atmosphere pollution':ab,ti OR 'atmospheric pollution':ab,ti OR 'polluted air':ab,ti OR 'polluted atmosphere':ab,ti OR 'pollution, air':ab,ti

58,699

#1

'air pollution'/exp OR 'air pollution'

**COCHARANE**

Air Quality; Pollution, Air; Air Pollutions

Environmental Pollutants, Air; Environmental Air Pollutants; Pollutants, Environmental Air; Air Environmental Pollutants; Air Pollutants, Environmental; Pollutants, Air Environmental; Air Pollutant; Pollutants, Air; Pollutant, Air

Allergic Rhinitis; Rhinitides, Allergic; Allergic Rhinitides

Animal; Animalia; Metazoa

Laboratory Mice; Laboratory Mouse; Mice, Laboratory; Mouse, Laboratory; Swiss Mice; Mice, Swiss; Mouse, Swiss; Swiss Mouse; Mouse; Mus; Mouse, House; House Mouse; Mus musculus; Mice, House; House Mice; Mus domesticus; Mus musculus domesticus; domesticus, Mus musculus

**PUBMED\Web of Science**

- Air Pollutions
- Pollution, Air
- Air Quality

(air pollution OR Air Pollutions OR Pollution, Air OR Air Quality OR ambient air pollution OR environmental pollution OR Polycyclic aromatic hydrocarbons OR particulate matter OR PM2.5 OR PM10 OR nitrogen dioxide OR NO2 OR NO OR ozone OR O3 OR sulfur dioxide OR SO2 OR carbon monoxide OR CO)

allergic rhinitis

- Allergic Rhinitides
- Rhinitides, Allergic
- Allergic Rhinitis
- Rhinitis, Allergic
- "allergic rhinitis" OR Rhinitides, Allergic OR Allergic Rhinitides OR Rhinitis, Allergic
- animal OR Animalia OR Metazoa OR mouse OR murine OR Mus OR Mice, Laboratory OR Laboratory Mice OR Mouse, Swiss OR Swiss Mouse OR Swiss Mice OR Mice, Swiss OR Mus domesticus OR Mus musculus domesticus OR domesticus, Mus musculus OR Mus musculus OR Mouse, House OR House Mouse OR Mice, House OR House Mice
- OR Mus domesticus
- Animal
- Animalia
- Metazoa
- Mus
- Mouse
- Mice, Laboratory
- Laboratory Mice
- Mouse, Laboratory
- Laboratory Mouse
- Mouse, Swiss
- Swiss Mouse
- Swiss Mice
- Mice, Swiss
- Mus domesticus
- Mus musculus domesticus
- domesticus, Mus musculus
- Mus musculus
- Mouse, House
- House Mouse
- Mice, House
- House Mice

(air pollution OR Air Pollutions OR Pollution, Air OR Air Quality OR ambient air pollution OR environmental pollution OR Polycyclic aromatic hydrocarbons OR particulate matter OR PM2.5 OR PM10 OR nitrogen dioxide OR NO2 OR NO OR ozone OR O3 OR sulfur dioxide OR SO2 OR carbon monoxide OR CO) AND ("allergic rhinitis" OR Rhinitides, Allergic OR Allergic Rhinitides OR Rhinitis, Allergic) AND ("inflammatory marker" OR "inflammatory mediator" OR cytokine OR interleukin OR "IL" OR tumor necrosis factor OR TNF OR TNF-alpha OR interferon OR IFN OR histamine OR leukotriene OR eosinophil OR eosinophilic OR eosinophils OR mast cells OR lymphocytes OR neutrophils OR macrophages OR nasal mucosa OR nasal secretions OR IgE OR CRP OR Fibrinogen OR NLRP3) AND (animal OR Animalia OR Metazoa OR mouse OR murine OR Mus OR Mice, Laboratory OR Laboratory Mice OR Mouse, Swiss OR Swiss Mouse OR Swiss Mice OR Mice, Swiss OR Mus domesticus OR Mus musculus domesticus OR domesticus, Mus musculus OR Mus musculus OR Mouse, House OR House Mouse OR Mice, House OR House Mice){, #205}
