# Supplementary material for: Systematic review and meta-analysis of the effects of air pollution exposure on nasal mucosal immune-inflammatory markers in experimental animal models of AR
Source: Front Pharmacol. 2026 Jul 16;17:1870023. doi: 10.3389/fphar.2026.1870023 (PMC13422168; doi:10.3389/fphar.2026.1870023)
Supplement: Supplementary file 1 [file Supplementaryfile1.zip › Supplementary file 1/Supplementary Table 10.docx]

**Table 10.**Publication bias

|  |  | **Begg's Test** | | **Egger's test** | |
| --- | --- | --- | --- | --- | --- |
| Indicator | n | Z | P | t | P |
| IgE | 4 | 0.68 | 0.4969 | 0.61 | 0.6059 |
| OVA-IgE | 12 | 3.02 | 0.0026 | 6.63 | <0.0001 |
| Neu | 4 | 0.00 | 1.000 | 0.45 | 0.696 |
| Eos | 12 | 1.67 | 0.0998 | 2.06 | 0.0666 |
| IL-4 | 8 | 1.98 | 0.0478 | 2.07 | 0.0839 |
| IL-5 | 7 | 1.35 | 0.1765 | 3.07 | 0.0277 |
| IL-13 | 7 | 2.55 | 0.0107 | 6.54 | 0.0012 |
| IL-17 | 3 | 1.57 | 0.1172 | 2.29 | 0.2623 |
| IFN-γ | 7 | 0.15 | 0.8806 | 0.45 | 0.6725 |
| NLRP3 | 3 | 1.57 | 0.1172 | 7.74 | 0.0818 |
| IL-1**β** | 3 | 0.52 | 0.6015 | 5.88 | 0.1073 |
| ZO-1 | 3 | -0.52 | 0.602 | -1.19 | 0.445 |
