# Supplementary material for: Systematic review and meta-analysis of the effects of air pollution exposure on nasal mucosal immune-inflammatory markers in experimental animal models of AR
Source: Front Pharmacol. 2026 Jul 16;17:1870023. doi: 10.3389/fphar.2026.1870023 (PMC13422168; doi:10.3389/fphar.2026.1870023)
Supplement: Supplementary file 1 [file Supplementaryfile1.zip › Supplementary file 1/Supplementary Table 11.docx]

| **Outcome** | **Main analysis** | **Alternative rule analysis** | **Direction changed?** | **Significance changed?** | **Interpretation** |
| --- | --- | --- | --- | --- | --- |
| Eosinophils | SMD = 2.38 (95% CI: 1.03-3.74) | SMD = 1.49 (95% CI: 0.55-2.44) | no | no | Conclusion retained; the effect size was attenuated under the alternative rule. |
| IL-4 | SMD = 2.42 (95% CI: 0.84-4.00) | SMD = 1.56 (95% CI: 0.40-2.73) | no | no | Conclusion retained; the effect size was attenuated under the alternative rule. |
| IL-5 | SMD = 3.68 (95% CI: 1.85-5.51) | SMD = 3.15 (95% CI: 1.48-4.82) | no | no | Conclusion retained; the effect size was attenuated under the alternative rule. |
| IL-13 | SMD = 4.60 (95% CI: 2.20-7.01) | SMD = 3.05 (95% CI: 1.27-4.83) | no | no | Conclusion retained; the effect size was attenuated under the alternative rule. |
| OVA-specific IgE | SMD = 3.54 (95% CI: 2.13-4.94) | SMD = 2.23 (95% CI: 1.25-3.21) | no | no | Conclusion retained; the effect size was attenuated under the alternative rule. |

**Table 11**
