# Supplementary material for: Systematic review and meta-analysis of the effects of air pollution exposure on nasal mucosal immune-inflammatory markers in experimental animal models of AR
Source: Front Pharmacol. 2026 Jul 16;17:1870023. doi: 10.3389/fphar.2026.1870023 (PMC13422168; doi:10.3389/fphar.2026.1870023)
Supplement: Supplementary file 1 [file Supplementaryfile1.zip › Supplementary file 1/Supplementary Table 12.docx]

| **Mechanistic domain** | **Directly measured markers** | **Evidence level** | **Recommended interpretation** |
| --- | --- | --- | --- |
| Type 2 inflammation | Eosinophils, IL-4, IL-5, IL-13, OVA-specific IgE | Relatively stronger | Most consistent signal; suitable for cautious biomarker discussion |
| Epithelial barrier disruption | ZO-1 | Limited | Biologically plausible; exploratory due to few studies |
| Inflammasome/innate activation | NLRP3, IL-1β | Limited | Preliminary mechanistic clue; causal sequence not proven |
| Th1 modulation | IFN-γ | Inconclusive | No consistent effect; avoid firm Th1 conclusions |
| Th17/alarmin pathways | IL-17, IL-25, IL-33 | Limited/exploratory | Needs additional targeted studies |

**Table 12**
