# Supplementary material for: Systematic review and meta-analysis of the effects of air pollution exposure on nasal mucosal immune-inflammatory markers in experimental animal models of AR
Source: Front Pharmacol. 2026 Jul 16;17:1870023. doi: 10.3389/fphar.2026.1870023 (PMC13422168; doi:10.3389/fphar.2026.1870023)
Supplement: Supplementary file 1 [file Supplementaryfile1.zip › Supplementary file 1/Supplementary Table 2.docx]

| **Element** | **Description** |
| --- | --- |
| **Population (P)** | Restricted to animal models of allergic rhinitis. |
| **Intervention (I)** | Exposure to one or more environmental air pollutants (e.g., PM2.5, PM10, O₃, DEP, SO₂, NO₂), administered via whole-body inhalation, intranasal instillation, or other relevant routes. |
| **Comparator (C)** | Control group not exposed to the target pollutants (typically clean air or solvent control). |
| **Outcomes (O)** | Primary outcomes include quantitative measurement of immune and inflammatory markers in nasal mucosal tissue, nasal lavage fluid, or serum. Key indicators include but are not limited to: Inflammatory cell counts (eosinophils, neutrophils, macrophages, lymphocytes); Cytokines (IL-1β, IL-4, IL-5, IL-13, IL-17, TNF-α, IFN-γ, IL-25, IL-33); Immunoglobulins (total IgE, ovalbumin-specific IgE); Epithelial barrier proteins (e.g., ZO-1); Inflammasome components (e.g., NLRP3). |
| **Timing (T)** | Duration of interventions ranged from 3 days to 42 days. |
| **Study Design (S)** | Published randomized controlled trials (RCTs) in animal models, employing double-blind or single-blind designs, with either parallel-group or crossover designs. |

**Table 2**
