# Supplementary material for: Systematic review and meta-analysis of the effects of air pollution exposure on nasal mucosal immune-inflammatory markers in experimental animal models of AR
Source: Front Pharmacol. 2026 Jul 16;17:1870023. doi: 10.3389/fphar.2026.1870023 (PMC13422168; doi:10.3389/fphar.2026.1870023)
Supplement: Supplementary file 1 [file Supplementaryfile1.zip › Supplementary file 1/Supplementary Table 3.docx]

**Table 3.** Characteristics of the studies included in the meta-analysis.

| **Author** | **Country** | **Year** | **Animal** | **Sex** | **Per-Arm Sample Sizes** | **Modeling method** | **Types of pollutants** | **Exposure routes** | **Concentration** | **Frequency** | **Duration** | **Outcome** | **Randomization** | **Blinding** |
| --- | --- | --- | --- | --- | --- | --- | --- | --- | --- | --- | --- | --- | --- | --- |
| Fukuoka, A | Japan | 2016 | BALB/c mice | Female | 3 | Ragweed pollen solution | DEP | Intranasal instillation | 20 μL | 1 times a week; 4 weeks | 4 days | ZO-1;EOS | Yes | Yes |
| Guo,Z.Q | China | 2017 | SD rats | Female | 8 | OVA solution | PM2.5 | Whole-body inhalation | 3000μg/m3 | Freq:4 times; days 1. 5. 10. 14 | 30 days | EOS；IFN-γ；ova-IgE | Yes | Unclear |
| Iijima,M. K | Japan | 2001 | Hartley guinea pigs | Male | 8/7 | OVA solution | O3 | Whole-body inhalation | 0.4 ppm | Freq: once a day; 7 days | 35 days | EOS;ova-IgE | Yes | Unclear |
| Iijima,M. K | Japan | 2004 | Hartley guinea pigs | Male | 8/7 | OVA solution | O3 | Whole-body inhalation | 0.6 ppm | Freq:6.5 days a week ，24 hours a day | 32.5 days | EOS;ova-IgE | Yes | Unclear |
| Jung,H. J | Korea | 2021 | BALB/c mice | Female | 10 | HDM solution | DEP | Intranasal instillation | 100μL | Freq: alternate days; 2 weeks | 10 days | IgE;EOS; IL-4；IL-13；IFN-；IL-17；IL-25；IL-33；TSLP | Yes | Yes |
| Li,J | China | 2021 | C57BL/6 mice | Female | 8 | OVA solution | PM2.5 | Intranasal instillation | 4.0 mg/kg | Freq: once a day; 3 weeks | 7 days | ova-IgE;NLRP3;IL-1β | Yes | Unclear |
| Li,J | China | 2024 | C57BL/6 mice | Female | 10 | OVA solution | O3 | Whole-body inhalation | 1ppm | Freq: alternate days; 2 weeks | 21 days | ova-IgE;ZO-1; | Yes | Unclear |
| Li, J | China | 2025 | C57BL/6 mice | Female | 10 | OVA solution | PM2.5 | Intranasal instillation | 4.0 mg/kg | Freq: once a day; 14 days | 7 days | ova-IgE;ZO-1;NLRP3;IL-1β | Yes | Unclear |
| Li,Y.J | China | 2019 | BALB/c mice | Male | 10 | OVA solution | PM2.5 | Whole-body inhalation | 125 μg/m3 | Freq: twice a week; for 4 weeks | 21 days | EOS;ova-IgE | Yes | Yes |
| Park,J.H | Korea | 2025 | BALB/c mice | Female | 3 | OVA solution | PM2.5 | Intranasal instillation | 10µg/m3 | Freq: 2 times; days 0.14 | 4 days | IgE;EOS;ova-IgE;IL-4；IL-13；IFN-；IL-17；Neu;Lym; | Yes | Yes |
| Piao,C. H | China | 2021 | BALB/c mice | Male | 6 | OVA solution | PM2.5 | Intranasal instillation | 20 μ L | Freq: 6 times; days 8.9.16.17.24.25 | 21 days | ova-IgE;Eos;IL-4；IL-5；IL-13;IFN-；IL-17;Neu;Mac | Yes | Unclear |
| Sun,N | China | 2021 | SD rats | Female | 10 | OVA solution | O3 | Whole-body inhalations | 2.0 ppm | Freq: 2 times; days 1.15 | 42 days | ova-IgE;;IL-4; IL-5; IL-13;IMac; Lym; Eos; Neu; IFN-γ | Yes | Yes |
| Sun,N | China | 2023 | SD rats | Female | 12 | OVA solution | O3 | Whole-body inhalation | 2.0 ppm | Freq: 1 times a week; 3weeks | 42 days | ova-IgE; | Yes | Yes |
| Wagner, J.G | USA | 2002 | Norwegian rats | Male | 6 | OVA solution | O3 | Whole-body inhalation | 0.5 ppm |  | 3 days | Eos; Neu; | Yes | Unclear |
| Wang,Y.L | China | 2017 | SD rats | Male | 15 | OVA solution | PM2.5 | Intranasal instillation | 0.20 mg/mL |  | 15 days | IgE;EOS;IL-4;IL-5;IL-33; IFN-γ; | Yes | Unclear |
| Ye,M.Y | China | 2022 | C57BL/6 mice | Female | 6 | HDM solution | SO2 | Whole-body inhalation | 150 mg/m^3^ |  | 30 days | IgE;EOS;IL-4;IL-5;IL-33; IFN-γ; | Yes | Yes |
| Zhang,C.H | China | 2025 | BALB/c mice | Female | 6 | OVA solution | PM2.5 | Whole-body inhalation | 72.15 µg/m³ |  | 42 days | IgE;IL-4;IL-5;IL-33; IFN-γ;TNF-a； | Yes | Unclear |
| Zhang, X.Y | China | 2023 | SD rats | Female | 10 | OVA solution | O3 | intraperitoneal injections | 2 ppm |  | 28 days | ova-IgE;;EOS;IL-4; IL-5;IL-13;NLRP3;IL-1β | Yes | Unclear |

T: experimental group; C: control group; HIS: Histamine; Mac: macrophages; Lym: lymphocytes; Neu,:neutrophils; Eos: eosinophils; IL: interleukin;

TNF-α: tumor necrosis factor-α; IFN- γ: interferoneinterferone-γ

PQ: Bra c p-quercetin covalent polymer; QU: Quercetin; QS: Quercetin; QI: Quercitrin,

DNCB: Dinitrochlorobenzene; DF: Dermatophagoides farina; CPE: crude PN extract; QGR:Quercetin-3-O-(200-gallate)-a-l-rhamnopyranoside
