# Supplementary material for: Systematic review and meta-analysis of the effects of air pollution exposure on nasal mucosal immune-inflammatory markers in experimental animal models of AR
Source: Front Pharmacol. 2026 Jul 16;17:1870023. doi: 10.3389/fphar.2026.1870023 (PMC13422168; doi:10.3389/fphar.2026.1870023)
Supplement: Supplementary file 1 [file Supplementaryfile1.zip › Supplementary file 1/Supplementary Table 4.docx]

**Table 4**

| **Author** | **Sequence generation Bias** | **Baseline characteristics Bias** | **Allocation concealment bias** | **Random Housing Bias** | **Blinding of Personnel Bias** | **Random outcome Assessment Bias** | **Blinding of outcome assessors bias** | **Incomplete data Reporting Bias** | **Selective outcome reporting Bias** | **Other potential confounding Biases** | **Score** |
| --- | --- | --- | --- | --- | --- | --- | --- | --- | --- | --- | --- |
| A.Fukuoka 2016 | Uncertain | Yes | Uncertain | uncertain | No | Uncertain | Yes | Yes | Yes | uncertain | 14 |
| Zhi-Qiang Guo 2017 | Uncertain | Yes | Uncertain | Uncertain | No | Uncertain | No | Yes | Yes | Uncertain | 11 |
| Mariko K Iijima 2004 | Uncertain | Yes | Uncertain | Uncertain | No | Uncertain | No | Yes | Yes | Uncertain | 11 |
| James G Wagner 2002 | Uncertain | Yes | Uncertain | Uncertain | No | Uncertain | No | Yes | Yes | Uncertain | 11 |
| Joo-Hoo Park 2025 | Uncertain | Yes | Uncertain | Uncertain | No | Uncertain | Yes | Yes | Yes | Uncertain | 14 |
| Hahn Jin Jung 2021 | Uncertain | Yes | Uncertain | Uncertain | Yes | Uncertain | Yes | Yes | Yes | Uncertain | 17 |
| Juan Li 2024 | Uncertain | Yes | Uncertain | Uncertain | No | Uncertain | No | Yes | Yes | Uncertain | 11 |
| Juan Li 2025 | Uncertain | Yes | Uncertain | Uncertain | No | Uncertain | No | Yes | Yes | Uncertain | 11 |
| Li, Juan 2021 | Uncertain | Yes | Uncertain | Uncertain | No | Uncertain | No | Yes | Yes | Uncertain | 11 |
| Li,Youjin 2019 | Uncertain | Yes | Uncertain | Uncertain | No | Uncertain | Yes | Yes | Yes | Uncertain | 14 |
| M K Iijima 2001 | Uncertain | Yes | Uncertain | Uncertain | No | Uncertain | No | Yes | Yes | Uncertain | 11 |
| Chun Hua Piao 2021 | Uncertain | Yes | Uncertain | Uncertain | No | Uncertain | Yes | Yes | Yes | Uncertain | 14 |
| Sun, Na 2023 | Uncertain | Yes | Uncertain | Uncertain | No | Uncertain | Yes | Yes | Yes | Uncertain | 14 |
| Sun, Na 2021 | Uncertain | Yes | Uncertain | Uncertain | No | Uncertain | Yes | Yes | Yes | Uncertain | 14 |
| Wang, Ya-Lin 2017 | Uncertain | Yes | Uncertain | Uncertain | No | Uncertain | No | Yes | Yes | Uncertain | 11 |
| Ye, Maoyu 2022 | Uncertain | Yes | Uncertain | Uncertain | No | Uncertain | Yes | Yes | Yes | Uncertain | 14 |
| Zhang, Xueyan 2023 | Uncertain | Yes | Uncertain | Uncertain | No | Uncertain | No | Yes | Yes | Uncertain | 11 |
| ChiHang Zhang 2025 | Uncertain | Yes | Uncertain | Uncertain | No | Uncertain | No | Yes | Yes | Uncertain | 11 |

| **Author** | **Sequence**  **generation**  **bias** | **Baseline**  **characteristics**  **bias** | **Allocation**  **concealme**  **nt bias** | **Random**  **housing**  **bias** | **Blinding of**  **personnel**  **bias** | **Random**  **outcome**  **assessment**  **bias** | **Blinding of**  **outcome**  **assessors bias** | **Incomplete**  **data**  **reporting**  **bias** | **Selective**  **outcome**  **reporting**  **bias** | **Other**  **potential**  **confounding**  **biases** | **Sco**  **re** |
| --- | --- | --- | --- | --- | --- | --- | --- | --- | --- | --- | --- |
| Enning Zhou | uncertain | yes | uncertain | uncertain | no | uncertain | no | yes | yes | yes | 11 |
