# Supplementary material for: Systematic review and meta-analysis of the effects of air pollution exposure on nasal mucosal immune-inflammatory markers in experimental animal models of AR
Source: Front Pharmacol. 2026 Jul 16;17:1870023. doi: 10.3389/fphar.2026.1870023 (PMC13422168; doi:10.3389/fphar.2026.1870023)
Supplement: Supplementary file 1 [file Supplementaryfile1.zip › Supplementary file 1/Supplementary Table 5.docx]

**Table 5 .**Subgroup analysis by pollutant indicated

| **Outcome** | **Subgroup** | **n(k)** | **N** | **I^2^** | **P(het)** | **SMD** | **95%CI** | **P(effect)** | **P(between)** |
| --- | --- | --- | --- | --- | --- | --- | --- | --- | --- |
| IgE | PM2.5 | 2 | 36 | 64.5% | 0.0931 | 5.06 | (-0.28,10.40) | 0.063 | 0.0803 |
|  | DEP | 1 | 20 | 0.0% | - | -1.10 | (-2.04,-0.15) | 0.023 |  |
|  | SO2 | 1 | 12 | 0.0% | - | -1.15 | (-2.38,0.10) | 0.070 |  |
|  |  |  |  |  |  |  |  |  |  |
| OVA-IgE | PM2.5 | 6 | 94 | 88.2% | <0.001 | 3.35 | (1.38,5.32) | 0.001 | 0.667 |
|  | O3 | 6 | 115 | 93.4% | <0.001 | 4.00 | (1.77,6.24) | <0.001 |  |
|  |  |  |  |  |  |  |  |  |  |
| Eos | DEP | 2 | 26 | 86.9% | 0.0057 | -1.46 | (-4.30,-1.37) | 0.312 | 0.0184 |
|  | PM2.5 | 5 | 84 | 86.1% | <0.0001 | 3.04 | (1.20,4.91) | 0.001 |  |
|  | O3 | 4 | 63 | 85.8% | 0.0001 | 3.03 | (1.03,5.04) | 0.003 |  |
|  | SO2 | 1 | 12 | 0.0% | - | 4.04 | (1.91,6.16) | <0.001 |  |
|  |  |  |  |  |  |  |  |  |  |
| Lym | PM2.5 | 1 | 3 | 0.0% | - | 10.28 | (2.66,17.89) | 0.008 | 0.0478 |
|  | O3 | 1 | 10 | 0.0% | - | 3.86 | (2.32,5.41) | <0.001 |  |
|  |  |  |  |  |  |  |  |  |  |
| Neu | O3 | 2 | 32 | 89.8% | 0.0017 | -0.23 | (-2.78,2.32) | 0.858 | 0.2916 |
|  | PM2.5 | 2 | 18 | 75.8% | 0.0422 | 2.31 | (-1.67,6.30) | 0.255 |  |
|  |  |  |  |  |  |  |  |  |  |
| IL-4 | PM2.5 | 5 | 76 | 84% | <0.0001 | 3.28 | (1.28,5.27) | 0.001 | <0.0001 |
|  | O3 | 1 | 20 | 0.0% | - | 2.16 | (1.03,3.29) | <0.001 |  |
|  | DEP | 1 | 20 | 0.0% | - | -1.08 | (-2.02,-0.13) | 0.026 |  |
|  | SO2 | 1 | 12 | 0.0% | - | 2.71 | (1.06,4.36) | 0.001 |  |
|  |  |  |  |  |  |  |  |  |  |
| IL-5 | PM2.5 | 4 | 60 | 74.1% | 0.0089 | 2.66 | (0.96,4.37) | 0.002 | 0.5799 |
|  | O3 | 2 | 20 | 96.6% | <0.0001 | 15.05 | (-10.72,40.82) | 0.252 |  |
|  | SO2 | 1 | 12 | 0.0% | - | 3.30 | (1.45,5.15) | <0.001 |  |
|  |  |  |  |  |  |  |  |  |  |
| IL-13 | PM2.5 | 3 | 40 | 86.6% | 0.0006 | 6.30 | (1.78,10.83) | 0.006 | 0.0004 |
|  | DEP | 1 | 20 | 0.0% | - | -0.59 | (-1.49,0.31) | 0.199 |  |
|  | O3 | 2 | 40 | 96% | <0.0001 | 8.87 | (-4.97,22.71) | 0.209 |  |
|  | SO2 | 1 | 12 | 0.0% | - | 2.17 | (0.28,3.66) | 0.004 |  |
|  |  |  |  |  |  |  |  |  |  |
| IFN-γ | PM2.5 | 4 | 64 | 95.6% | <0.001 | 0.29 | (-4.36,4.93) | 0.903 | <0.0001 |
|  | DEP | 1 | 20 | 0.0% | - | -0.31 | (-1.19,0.58) | 0.497 |  |
|  | O3 | 1 | 20 | 0.0% | - | -1.98 | (-3.07,-0.89) | <0.001 |  |
|  | SO2 | 1 | 12 | 0.0% | - | 12.76 | (6.97,18.54) | <0.001 |  |
|  |  |  |  |  |  |  |  |  |  |
| IL-17 | PM2.5 | 2 | 18 | 84% | 0.0123 | 4.95 | (-5.20,15.10) | 0.339 | 0.5815 |
|  | SO2 | 1 | 12 | 0.0% | - | 2.07 | (0.61,3.53) | 0.005 |  |
|  |  |  |  |  |  |  |  |  |  |
| NLRP3 | O3 | 1 | 20 | 0.0% | - | 3.86 | (2.32,5.40) | <0.001 | 0.7440 |
|  | PM2.5 | 2 | 36 | 85.6% | 0.0084 | 3.31 | (0.43,6.20) | 0.025 |  |
|  |  |  |  |  |  |  |  |  |  |
| IL-1β | PM2.5 | 2 | 36 | 93.1% | 0.0001 | 5.02 | (-0.87,10.92) | 0.095 | 0.4031 |
|  | SO2 | 1 | 20 | 0.0% | - | 2.46 | (1.27,3.65) | <0.001 |  |
|  |  |  |  |  |  |  |  |  |  |
| ZO-1 | DEP | 1 | 6 | 0.0% | - | -3.41 | (-6.36,-0.47) | 0.023 | 0.0014 |
|  | O3 | 1 | 20 | 0.0% | - | -1.91 | (-2.99,-0.83) | <0.001 |  |
|  | PM2.5 | 1 | 20 | 0.0% | - | -6.70 | (-9.08,-4.33) | <0.001 |  |
|  |  |  |  |  |  |  |  |  |  |
| IL-33 | DEP | 1 | 20 | 0.0% | - | 0.49 | (-0,40,1.38) | 0.281 | 0.0055 |
|  | PM2.5 | 1 | 30 | 0.0% | - | 2.33 | (1.39,3.27) | <0.001 |  |
|  |  |  |  |  |  |  |  |  |  |

n (k) = number of studies; N = total number of animals.
