# Supplementary material for: Systematic review and meta-analysis of the effects of air pollution exposure on nasal mucosal immune-inflammatory markers in experimental animal models of AR
Source: Front Pharmacol. 2026 Jul 16;17:1870023. doi: 10.3389/fphar.2026.1870023 (PMC13422168; doi:10.3389/fphar.2026.1870023)
Supplement: Supplementary file 1 [file Supplementaryfile1.zip › Supplementary file 1/Supplementary Table 6.docx]

**Table 6.**Subgroup analysis by exposure route indicated

| **Outcome** | **Subgroup** | **n(k)** | **N** | **I^2^** | **P(het)** | **SMD** | **95%CI** | **P(effect)** | **P(between)** |
| --- | --- | --- | --- | --- | --- | --- | --- | --- | --- |
| IgE | **Whole-Body Inhalation** | 1 | 12 | 0.0% | - | -1.15 | (-2.39, 0.10) | 0.070 | 0.0818 |
|  | **Nasal Instillation** | 3 | 56 | 94.8% | <0.0001 | 2.51 | (-1.42, 6.45) | 0.210 |  |
|  |  |  |  |  |  |  |  |  |  |
| OVA-IgE | **Whole-Body Inhalation** | 8 | 151 | 92.4% | <0.0001 | 3.97 | (2.13, 5.80) | <0.001 | 0.566 |
|  | **Nasal Instillation** | 4 | 58 | 89% | <0.0001 | 3.01 | ( 0.32;,5.71) | 0.028 |  |
|  |  |  |  |  |  |  |  |  |  |
| Eos | **Nasal Instillation** | 5 | 74 | 93.3% | <0.0001 | 1.24 | (-1.43, 3.91) | 0.363 | 0.2213 |
|  | **Whole-Body Inhalation** | 7 | 111 | 85.2% | <0.0001 | 3.14 | (1.66, 4.63) | <0.001 |  |
|  |  |  |  |  |  |  |  |  |  |
| Lym | **Nasal Instillation** | 1 | 3 | 0.0% | - | 10.28 | (2.66,17.89) | 0.008 | 0.0478 |
|  | Whole-Body Inhalation | 1 | 10 | 0.0% | - | 3.86 | (2.32,5.41) | <0.001 |  |
|  |  |  |  |  |  |  |  |  |  |
| Neu | **Whole-Body Inhalation** | 2 | 32 | 89.8% | 0.0017 | -0.23 | (-2.78,2.32) | 0.858 | 0.2916 |
|  | **Nasal Instillation** | 2 | 18 | 75.8% | 0.0422 | 2.31 | (-1.67,6.30) | 0.255 |  |
|  |  |  |  |  |  |  |  |  |  |
| IL-4 | **Whole-Body Inhalation** | 4 | 60 | 44.7% | <0.0001 | 2.99 | (1.91, 4.07) | <0.001 | 0.3969 |
|  | **Nasal Instillation** | 4 | 68 | 92.8% | 0.1430 | 1.75 | (-0.92, 4.41) | 0.199 |  |
|  |  |  |  |  |  |  |  |  |  |
| IL-5 | **Whole-Body Inhalation** | 3 | 44 | 82.8% | <0.0001 | 4.80 | (1.67, 7.93) | 0.003 | 0.3968 |
|  | **Nasal Instillation** | 3 | 48 | 89.9% | 0.0030 | 3.03 | (0.39, 5.68) | 0.025 |  |
|  |  |  |  |  |  |  |  |  |  |
| IL-13 | **Whole-Body Inhalation** | 5 | 80 | 88.7% | <0.0001 | 4.84 | (2.31, 7.37) | <0.001 | 0.9476 |
|  | **Nasal Instillation** | 2 | 32 | 94.9% | <0.0001 | 4.48 | (-5.93, 14.90) | 0.399 |  |
|  |  |  |  |  |  |  |  |  |  |
| IFN-γ | **Whole-Body Inhalation** | 4 | 60 | 91.6% | <0.0001 | -0.73 | (-4.11, 2.65) | 0.672 | 0.1693 |
|  | **Nasal Instillation** | 3 | 56 | 93% | <0.0001 | 2.59 | (-0.73, 5.92) | 0.126 |  |
|  |  |  |  |  |  |  |  |  |  |
| IL-17 | **Whole-Body Inhalation** | 1 | 12 | 0.0% | - | 2.07 | (0.61,3.53) | 0.005 | 0.5815 |
|  | **Nasal Instillation** | 2 | 18 | 85.3% | 0.0123 | 4.95 | (-5.20,15.10) | 0.339 |  |
|  |  |  |  |  |  |  |  |  |  |
| NLRP3 | **Whole-Body Inhalation** | 1 | 20 | 0.0% | - | 3.86 | (2.32,5.40) | <0.001 | 0.7440 |
|  | **Nasal Instillation** | 2 | 36 | 85.6% | 0.0084 | 3.31 | (0.43,6.20) | 0.025 |  |
|  |  |  |  |  |  |  |  |  |  |
| IL-1β | **Nasal Instillation** | 2 | 36 | 93.1% | 0.0001 | 5.02 | (-0.87,10.92) | 0.095 | 0.4031 |
|  | **Whole-Body Inhalation** | 1 | 20 | 0.0% | - | 2.46 | (1.27,3.65) | <0.001 |  |
|  |  |  |  |  |  |  |  |  |  |
| ZO-1 | **Nasal Instillation** | 2 | 26 | 65.6% | 0.08 | -5.18 | (-8.39, -1.96) | 0.002 | 0.0591 |
|  | **Whole-Body Inhalation** | 1 | 20 | 0% | - | -1.91 | (-2.99,-0.83) | <0.001 |  |
|  |  |  |  |  |  |  |  |  |  |
| IL-33 | **Nasal Instillation** | 2 | 50 | 87% | - | 1.40 | (-0.40,3.20) | 0.127 | - |
|  |  |  |  |  |  |  |  |  |  |

n (k) = number of studies; N = total number of animals.
