# Supplementary material for: Systematic review and meta-analysis of the effects of air pollution exposure on nasal mucosal immune-inflammatory markers in experimental animal models of AR
Source: Front Pharmacol. 2026 Jul 16;17:1870023. doi: 10.3389/fphar.2026.1870023 (PMC13422168; doi:10.3389/fphar.2026.1870023)
Supplement: Supplementary file 1 [file Supplementaryfile1.zip › Supplementary file 1/Supplementary Table 7.docx]

**Table 7 .**Subgroup analysis by exposure time indicated

| **Outcome** | **Subgroup** | **n(k)** | **N** | **I^2^** | **P(het)** | **SMD** | **95%CI** | **P(effect)** | **P(between)** |
| --- | --- | --- | --- | --- | --- | --- | --- | --- | --- |
| IgE | Medium-term | 3 | 62 | 94.9% | <0.0001 | 0.30 | (-2.49, 3.09) | 0.009 | 0.0193 |
|  | Short-term | 1 | 6 | 0.0% | - | 8.92 | (2.26, 15.58) | 0.833 |  |
|  |  |  |  |  |  |  |  |  |  |
| OVA-IgE | Medium-term | 5 | 88 | 75.6% | 0.0026 | 2.38 | (1.20, 3.57) | <0.001 | 0.137 |
|  | Long-term | 4 | 75 | 96.7% | <0.0001 | 6.55 | (2.34,10.76) | 0.002 |  |
|  | Short-term | 3 | 46 | 92.6% | <0.0001 | 4.52 | (-0.47, 9.52) | 0.076 |  |
|  |  |  |  |  |  |  |  |  |  |
| Eos | Short-term | 5 | 44 | 73.6% | 0.0044 | 2.04 | (0.53, 3.55) | 0.008 | 0.6873 |
|  | Medium-term | 4 | 90 | 95.7% | <0.0001 | 1.93 | (-1.54, 5.40) | 0.275 |  |
|  | Long-term | 3 | 51 | 90.4% | <0.0001 | 3.41 | (0.59, 6.22) | 0.018 |  |
|  |  |  |  |  |  |  |  |  |  |
| Lym | Short-term | 1 | 3 | 0.0% | - | 10.28 | (2.66,17.89) | 0.008 | 0.0478 |
|  | Long-term | 1 | 10 | 0.0% | - | 3.86 | (2.32,5.41) | <0.001 |  |
|  |  |  |  |  |  |  |  |  |  |
| Neu | Short-term | 2 | 18 | 89.5% | 0.0020 | 1.35 | (-4.89, 7.59) | 0.671 | 0.880 |
|  | Medium-term | 1 | 12 | 0.0% | - | 0.65 | (-0.51, 1.82) | 0.272 |  |
|  | Long-term | 1 | 20 | 0.0% | - | 1.02 | (0.09,1.96) | 0.033 |  |
|  |  |  |  |  |  |  |  |  |  |
| IL-4 | Medium-term | 6 | 110 | 91.3% | <0.0001 | 2.05 | (0.30, 3.81) | 0.022 | 0.3549 |
|  | Short-term | 2 | 18 | 72.5% | 0.0566 | 5.56 | (-1.66, 12.78) | 0.131 |  |
|  |  |  |  |  |  |  |  |  |  |
| IL-5 | Medium-term | 4 | 37 | 56.6% | 0.0750 | 2.35 | (1.57, 3.11) | <0.001 | <0.0001 |
|  | Short-term | 2 | 9 | 73.8% | 0.0506 | 6.98 | (3.86, 22.19) | 0.139 |  |
|  | Long-term | 1 | 10 | 0.0% | - | 28.64 | (19.17, 38.11) | <0.001 |  |
|  |  |  |  |  |  |  |  |  |  |
| IL-13 | Medium-term | 5 | 92 | 90.5% | <0.001 | 3.61 | (1.03, 6.20) | 0.006 | <0.0001 |
|  | Short-term | 1 | 6 | 0.0% | - | 2.17 | (0.68, 3.66) | 0.004 |  |
|  | Long-term | 1 | 10 | 0.0% | - | 16.19 | (10.79,21.59) | <0.0001 |  |
|  |  |  |  |  |  |  |  |  |  |
| IFN-γ | Medium-term | 4 | 90 | 95.2% | <0.001 | -1.18 | (-4.17, 1.80) | 0.436 | <0.0001 |
|  | Short-term | 2 | 6 | 0.0% | - | 11.18 | (6.79,15.57) | <0.001 |  |
|  | Long-term | 1 | 20 | 0.0% | - | -1.98 | (-3.07,-0.89) | <0.001 |  |
|  |  |  |  |  |  |  |  |  |  |
| IL-17 | Medium-term | 2 | 24 | 62.6% | 0.1018 | 1.23 | (-0.29, 2.74) | 0.114 | 0.0205 |
|  | Short-term | 1 | 6 | 0.0% | - | 11.00 | (2.87, 19.12) | 0.008 |  |
|  |  |  |  |  |  |  |  |  |  |
| NLRP3 | Short-term | 2 | 36 | 85.6% | 0.0084 | 3.31 | (0.43,6.20) | 0.025 | 0.7440 |
|  | Medium-term | 1 | 20 | 0.0% | - | 3.86 | (2.32,5.40) | <0.001 |  |
|  |  |  |  |  |  |  |  |  |  |
| IL-1β | Short-term | 2 | 36 | 93.1% | 0.0001 | 5.02 | (-0.87,10.92) | 0.095 | 0.4031 |
|  | Medium-term | 1 | 20 | 0.0% | - | 2.46 | (1.27,3.65) | <0.001 |  |
|  |  |  |  |  |  |  |  |  |  |
| ZO-1 | Short-term | 2 | 26 | 65.6% | 0.08 | -5.18 | (-8.39, -1.96) | 0.002 | 0.0591 |
|  | Medium-term | 1 | 20 | 0% | - | -1.91 | (-2.99,-0.83) | <0.001 |  |
|  |  |  |  |  |  |  |  |  |  |
| IL-33 | Medium-term | 2 | 50 | 87% | - | 1.40 | (-0.40,3.20) | 0.127 | - |
|  |  |  |  |  |  |  |  |  |  |

n (k) = number of studies; N = total number of animals.
