# Supplementary material for: Systematic review and meta-analysis of the effects of air pollution exposure on nasal mucosal immune-inflammatory markers in experimental animal models of AR
Source: Front Pharmacol. 2026 Jul 16;17:1870023. doi: 10.3389/fphar.2026.1870023 (PMC13422168; doi:10.3389/fphar.2026.1870023)
Supplement: Supplementary file 1 [file Supplementaryfile1.zip › Supplementary file 1/Supplementary Table 8.docx]

**Table 8 .**Subgroup analysis by animal species indicated

| **Outcome** | **Subgroup** | **n(k)** | **N** | **I^2^** | **P(het)** | **SMD** | **95%CI** | **P(effect)** | **P(between)** |
| --- | --- | --- | --- | --- | --- | --- | --- | --- | --- |
| IgE | Rat | 1 | 30 | 0.0% | - | 3.14 | (2.05, 4.23) | <0.001 | 0.0032 |
|  | Mouse | 3 | 38 | 76.8% | 0.0134 | -0.32 | (-2.36, 1.71) | 0.754 |  |
|  |  |  |  |  |  |  |  |  |  |
| OVA-IgE | Mouse | 6 | 98 | 82.9% | <0.0001 | 2.14 | (0.77,3.52) | 0.002 | **0.001** |
|  | Rat | 4 | 80 | 94.1% | <0.0001 | 9.48 | (3.54,15.43) | 0.002 |  |
|  | Guinea Pig | 2 | 31 | 0.0% | 0.4512 | 0.32 | **(-0.39-1.04)** | 0.373 |  |
|  |  |  |  |  |  |  |  |  |  |
| Eos | Mouse | 6 | 76 | 85.9% | <0.0001 | 1.03 | (-0.81, 2.88) | 0.273 | **0.0103** |
|  | Rat | 4 | 78 | 94.6% | 0.0013 | 4.90 | (2.73, 7.07) | <0.001 |  |
|  | Guinea Pig | 2 | 31 | 0% | 0.8422 | 1.47 | (0.66, 2.28) | <0.001 |  |
|  |  |  |  |  |  |  |  |  |  |
| Lym | Mouse | 1 | 3 | 0.0% | - | 10.28 | (2.66,17.89) | 0.008 | 0.0478 |
|  | Rat | 1 | 10 | 0.0% | - | 3.86 | (2.32,5.41) | <0.001 |  |
|  |  |  |  |  |  |  |  |  |  |
| Neu | Rat | 2 | 32 | 89.8% | 0.0017 | -0.23 | (-2.78,2.32) | 0.858 | 0.2916 |
|  | Mouse | 2 | 18 | 75.8% | 0.0422 | 2.31 | (-1.67,6.30) | 0.255 |  |
|  |  |  |  |  |  |  |  |  |  |
| IL-4 | Rat | 3 | 66 | 67.2% | 0.0476 | 3.32 | (1.92, 4.73) | <0.001 | 0.1901 |
|  | Mouse | 5 | 62 | 87.4% | <0.0001 | 1.68 | (-0.35, 3.70) | 0.105 |  |
|  |  |  |  |  |  |  |  |  |  |
| IL-5 | Rat | 3 | 50 | 93.3% | <0.0001 | 6.27 | (2.19, 10.34) | 0.003 | **0.0993** |
|  | Mouse | 4 | 42 | 66.3% | 0.0307 | 2.55 | (0.82, 4.27) | 0.004 |  |
|  |  |  |  |  |  |  |  |  |  |
| IL-13 | Rat | 3 | 56 | 94.1% | <0.0001 | 8.06 | (1.36, 14.76) | 0.018 | 0.1468 |
|  | Mouse | 4 | 56 | 90.8% | <0.0001 | 2.72 | (0.04, 5.40) | 0.046 |  |
|  |  |  |  |  |  |  |  |  |  |
| IFN-γ | Rat | 3 | 66 | 97% | <0.0001 | -1.57 | (-6.23, 3.09) | 0.509 | 0.1307 |
|  | Mouse | 4 | 50 | 90.7% | <0.0001 | 2.97 | (-0.64, 6.57) | 0.107 |  |
|  |  |  |  |  |  |  |  |  |  |
| 1L-17 | Mouse | 3 | 30 | 75.9% | - | 1.99 | (-0.39,4.36) | 0.1047 | - |
|  |  |  |  |  |  |  |  |  |  |
| NLRP3 | Rat | 1 | 20 | 0.0% | - | 3.86 | (2.32,5.40) | <0.001 | 0.7440 |
|  | Mouse | 2 | 36 | 85.6% | 0.0084 | 3.31 | (0.43,6.20) | 0.025 |  |
|  |  |  |  |  |  |  |  |  |  |
| IL-1β | Mouse | 2 | 36 | 93.1% | 0.0001 | 5.02 | (-0.87,10.92) | 0.095 | 0.4031 |
|  | Rat | 1 | 20 | 0.0% | - | 2.46 | (1.27,3.65) | <0.001 |  |
|  |  |  |  |  |  |  |  |  |  |
| ZO-1 | Mouse | 3 | 46 | 85% | - | -3.90 | (-6.96,-0.85) | 0.01 | - |
|  |  |  |  |  |  |  |  |  |  |
| IL-33 | Mouse | 1 | 20 | 0.0% | - | 0.49 | (-0,40,1.38) | 0.281 | 0.0055 |
|  | Rat | 1 | 30 | 0.0% | - | 2.33 | (1.39,3.27) | <0.001 |  |
|  |  |  |  |  |  |  |  |  |  |

n (k) = number of studies; N = total number of animals.
