# Supplementary material for: Systematic review and meta-analysis of the effects of air pollution exposure on nasal mucosal immune-inflammatory markers in experimental animal models of AR
Source: Front Pharmacol. 2026 Jul 16;17:1870023. doi: 10.3389/fphar.2026.1870023 (PMC13422168; doi:10.3389/fphar.2026.1870023)
Supplement: Supplementary file 1 [file Supplementaryfile1.zip › Supplementary file 1/Supplementary Table 9.docx]

**Table 9 .**Subgroup analysis by pollutant×duration indicated

| **Outcome** | **Subgroup** | **n(k)** | **N** | **I^2^** | **P(het)** | **SMD** | **95%CI** | **P(effect)** | **P(between)** |
| --- | --- | --- | --- | --- | --- | --- | --- | --- | --- |
| IgE | **PM2.5×Short-term** | 1 | 6 | 0.0% | - | 8.92 | (2.26, 15.58) | 0.009 | <0.0001 |
|  | **PM2.5×Medium-term** | 1 | 30 | 0.0% | - | 3.14 | (2.05, 4.23) | <0.001 |  |
|  | **DEP×Medium-term** | 1 | 20 | 0.0% | - | -1.10 | (-2.04, -0.15) | 0.023 |  |
|  | **SO2×Medium-term** | 1 | 12 | 0.0% | - | -1.15 | (-2.39, 0.10) | 0.070 |  |
|  |  |  |  |  |  |  |  |  |  |
| OVA-IgE | **PM2.5×Short-term** | 3 | 46 | 92.6% | < 0.0001 | 4.52 | (-0.47,9.52) | 0.076 | **0.134** |
|  | **PM2.5×Medium-term** | 3 | 48 | 82.9% | 0.0029 | 3.12 | (0.92,5.31) | 0.005 |  |
|  | **O3×Medium-term** | 2 | 40 | 68.7% | 0.0737 | 1.76 | (0.39,3.13) | 0.012 |  |
|  | **O3×Long-term** | 4 | 75 | 95.7% | < 0.0001 | 6.55 | (2.34,10.76) | 0.002 |  |
|  |  |  |  |  |  |  |  |  |  |
| Eos | **PM2.5×Short-term** | 2 | 26 | 76.4% | 0.0397 | 2.66 | (-1.71,7.03) | 0.232 | <0.0001 |
|  | **PM2.5×Medium-term** | 3 | 58 | 87.6% | 0.0003 | 3.48 | (0.97,5.99) | 0.007 |  |
|  | **O3×Short-term** | 1 | 12 | 0.00% | - | 2.44 | (0.87，4.01) | 0.002 |  |
|  | **O3×Long-term** | 3 | 51 | 90.4% | <0.0001 | 3.41 | (0.59,6.22) | 0.018 |  |
|  | **DEP×Medium-term** | 1 | 20 | 0.0% | - | -2.87 | (-4.16,-1.58) | <0.001 |  |
|  | **DEP×Short-term** | 1 | 6 | 0.0% | - | 0.03 | (-1.57,1.63) | 0.975 |  |
|  | **SO2×Short-term** | 1 | 12 | 0.0% | - | 4.04 | (1.91,6.16) | <0.001 |  |
|  |  |  |  |  |  |  |  |  |  |
| Lym | **PM2.5×**Short-term | 1 | 3 | 0.0% | - | 10.28 | (2.66,17.89) | 0.008 | 0.0478 |
|  | **O3×**Long-term | 1 | 10 | 0.0% | - | 3.86 | (2.32,5.41) | <0.001 |  |
|  |  |  |  |  |  |  |  |  |  |
| Neu | **O3×Short-term** | 1 | 12 | 0.0% | - | -1.58 | (-2.91,-0.25) | 0.020 | 0.0015 |
|  | **PM2.5×Short-term** | 1 | 20 | 0.0% | - | 4.81 | (0.98, 8.64) | 0.014 |  |
|  | **PM2.5×Medium-term** | 1 | 12 | 0.0% | - | 0.65 | (-0.51,1.82) | 0.272 |  |
|  | **O3×Long-term** | 1 | 6 | 0.0% | - | 1.02 | (0.09,1.96) | 0.033 |  |
|  |  |  |  |  |  |  |  |  |  |
| IL-4 | **PM2.5×Medium-term** | 4 | 70 | 85.8% | <0.001 | 2.86 | (0.93,4.79) | 0.004 | <0.0001 |
|  | **PM2.5×Short-term** | 1 | 6 | 0.0% | - | 10.32 | (2.67,17.96) | 0.008 |  |
|  | **SO2×Short-term** | 1 | 12 | 0.0% | - | 2.71 | (1.06,4.36) | 0.001 |  |
|  | **DEP×Medium-term** | 1 | 20 | 0.0% | - | -1.08 | (-2.02,-0.13) | 0.026 |  |
|  | **O3×Medium-term** | 1 | 20 | 0.0% | - | 2.16 | (1.03,3.29) | <0.001 |  |
|  |  |  |  |  |  |  |  |  |  |
| IL-5 | **PM2.5×Short-term** | 1 | 6 | 0.0% | - | 13.02 | (3.45,22.59) | 0.008 | <0.0001 |
|  | **PM2.5×Medium-term** | 3 | 54 | 71.2% | 0.03 | 2.35 | (0.95,3.76) | 0.001 |  |
|  | **SO2×Short-term** | 1 | 12 | 0.0% | - | 3.30 | (1.45,5.15) | <0.001 |  |
|  | **O3×Medium-term** | 1 | 10 | 0.0% | - | 2.33 | (1.17,3.50) | <0.001 |  |
|  | **O3×Long-term** | 1 | 10 | 0.0% | - | 28.64 | (19.17, 38.11) | <0.001 |  |
|  |  |  |  |  |  |  |  |  |  |
| IL-13 | **PM2.5×Medium-term** | 3 | 40 | 86.6% | 0.0006 | 6.30 | (1.78,10.83) | 0.006 | <0.0001 |
|  | **SO2×Short-term** | 1 | 12 | 0.0% | - | 2.17 | (0.68,3.66) | 0.004 |  |
|  | **O3×Long-term** | 1 | 20 | 0.0% | - | 16.19 | (10.79,21.59) | <0.001 |  |
|  | **O3×Medium-term** | 1 | 20 | 0.0% | - | 2.06 | (0.95,3.17) | <0.001 |  |
|  | **DEP×Medium-term** | 1 | 20 | 0.0% | - | -0.59 | (-1.49,0.31) | 0.199 |  |
|  |  |  |  |  |  |  |  |  |  |
| IFN-γ | **PM2.5×Medium-term** | 3 | 58 | 96.7% | 0.001 | -1.61 | (-6.61,3.40) | 0.530 | <0.0001 |
|  | **PM2.5×Short-term** | 1 | 6 | 0.0% | - | 9.04 | (2.30,15.78) | 0.009 |  |
|  | **SO2×Short-term** | 1 | 12 | 0.0% | - | 12.76 | (6.97,18.54) | <0.001 |  |
|  | **O3×Long-term** | 1 | 20 | 0.0% | - | -1.98 | (-3.07,-0.89) | <0.001 |  |
|  | **DEP×Medium-term** | 1 | 20 | 0.0% | - | -0.31 | (-1.19,0.58) | 0.497 |  |
|  |  |  |  |  |  |  |  |  |  |
| IL-17 | **PM2.5×Short-term** | 1 | 6 | 0.0% | - | 11.00 | (2.87, 19.12) | 0.008 | 0.0159 |
|  | **PM2.5×Medium-term** | 1 | 12 | 0.0% | - | 0.52 | (-0.64,1.67) | 0.381 |  |
|  | **SO2×Medium-term** | 1 | 12 | 0.0% | - | 2.07 | (0.61,3.53) | 0.005 |  |
|  |  |  |  |  |  |  |  |  |  |
| NLRP3 | **PM2.5×Short-term** | 2 | 36 | 85.6% | 0.0084 | 3.31 | (0.43,6.20) | 0.025 | 0.7440 |
|  | **O3×Medium-term** | 1 | 20 | 0.0% | - | 3.86 | (2.32,5.40) | <0.001 |  |
|  |  |  |  |  |  |  |  |  |  |
| IL-1β | **PM2.5×Short-term** | 2 | 36 | 93.1% | 0.0001 | 5.02 | (-0.87,10.92) | 0.095 | 0.4031 |
|  | **O3× Medium-term** | 1 | 20 | 0.0% | - | 2.46 | (1.27,3.65) | <0.001 |  |
|  |  |  |  |  |  |  |  |  |  |
| ZO-1 | **DEP×Short-term** | 1 | 6 | 0.0% | - | -3.41 | (-6.36,-0.47) | 0.023 | 0.0014 |
|  | **O3×Medium-term** | 1 | 20 | 0.0% | - | -1.91 | (-2.99,-0.83) | <0.001 |  |
|  | **PM2.5×Short-term** | 1 | 20 | 0.0% | - | -6.70 | (-9.08,-4.33) | <0.001 |  |
|  |  |  |  |  |  |  |  |  |  |
| IL-33 | **PM2.5×**Medium-term | 1 | 30 | 0.0% | - | 0.49 | (-0,40,1.38) | 0.281 | 0.0055 |
|  | DEP**×**Medium-term | 1 | 20 | 0.0% | - | 2.33 | (1.39,3.27) | <0.001 |  |
|  |  |  |  |  |  |  |  |  |  |

n (k) = number of studies; N = total number of animals.
